# An Approximation to the Adaptive Exponential Integrate-and-Fire Neuron Model Allows Fast and Predictive Fitting to Physiological Data

**DOI:** 10.3389/fncom.2012.00062

**Published:** 2012-09-06

**Authors:** Loreen Hertäg, Joachim Hass, Tatiana Golovko, Daniel Durstewitz

**Affiliations:** ^1^Bernstein-Center for Computational Neuroscience, Central Institute of Mental Health, Psychiatry, Medical Faculty Mannheim of Heidelberg UniversityMannheim, Germany

**Keywords:** pyramidal cells, interneurons, *f*–*I* curve, adaptation, spike timing, temporal coding, prefrontal cortex

## Abstract

For large-scale network simulations, it is often desirable to have computationally tractable, yet in a defined sense still physiologically valid neuron models. In particular, these models should be able to reproduce physiological measurements, ideally in a predictive sense, and under different input regimes in which neurons may operate *in vivo*. Here we present an approach to parameter estimation for a simple spiking neuron model mainly based on standard *f*–*I* curves obtained from *in vitro* recordings. Such recordings are routinely obtained in standard protocols and assess a neuron’s response under a wide range of mean-input currents. Our fitting procedure makes use of closed-form expressions for the firing rate derived from an approximation to the adaptive exponential integrate-and-fire (AdEx) model. The resulting fitting process is simple and about two orders of magnitude faster compared to methods based on numerical integration of the differential equations. We probe this method on different cell types recorded from rodent prefrontal cortex. After fitting to the *f*–*I* current-clamp data, the model cells are tested on completely different sets of recordings obtained by fluctuating (“*in vivo*-like”) input currents. For a wide range of different input regimes, cell types, and cortical layers, the model could predict spike times on these test traces quite accurately within the bounds of physiological reliability, although no information from these distinct test sets was used for model fitting. Further analyses delineated some of the empirical factors constraining model fitting and the model’s generalization performance. An even simpler adaptive LIF neuron was also examined in this context. Hence, we have developed a “high-throughput” model fitting procedure which is simple and fast, with good prediction performance, and which relies only on firing rate information and standard physiological data widely and easily available.

## 1 Introduction

In recent years there has been a growing interest in large-scale neuronal network simulations (Traub et al., 1988, 2005; Whittington et al., 2000; Markram, 2006; Izhikevich and Edelman, 2008; Lansner, 2009; Lundqvist et al., 2010) that capture the cellular heterogeneity observed in real cortical tissue (Binzegger et al., 2004; Markram et al., 2004; Wang et al., 2006; Thomson and Lamy, 2007) and model interactions between many diverse cortical and subcortical brain structures (Lansner et al., 2003; Izhikevich and Edelman, 2008). The increasing desire to model such systems at a high level of physiological realism which takes into account the diversity and variation in neuronal cell types is, however, in conflict with the computational feasibility and “analytical” tractability of such models. Since in many situations synaptic inputs to a neuron with the same kinetics can be lumped into single “super-synapses” (e.g., Durstewitz and Gabriel, 2007), such that the number of synaptic equations to be solved scales linearly with the number of neurons, often the computational burden associated with the cellular models is the more serious bottleneck in network simulations. Moreover, if cell diversity is an explicit issue in itself and large pools of physiological cell data are available, even the time required for fitting cell models to all the different cell types recorded can become a considerable temporal constraint.

Single neuron models of very different degrees of complexity have been developed over the last decades to study neural functions. On one side, detailed multi-compartmental biophysically meaningful models can often reproduce voltage traces of their experimental counterparts to almost arbitrary degree (Traub et al., 1991; De Schutter and Bower, 1994; Jaeger et al., 1997; Poirazi and Mel, 2001; Prinz et al., 2003; Druckmann et al., 2007, 2011; Moyer et al., 2007), and potentially provide a deep understanding of the underlying biophysical mechanisms and functional role of the cellular morphology (e.g., Mainen and Sejnowski, 1996; Poirazi and Mel, 2001; Shu et al., 2006; Durstewitz and Gabriel, 2007). However, because of their large number of parameters, fitting such single-cell models to electrophysiological observations is often a slow and tedious procedure which may also run into the risk of serious over-fitting: Different parameter configurations may result in similarly good fits of a given “training set” (Prinz et al., 2004), thus it is not clear how these models would perform on data that was not explicitly used to optimize the parameters.

Partly for these reasons, but also for speeding up large-scale network simulations and mathematical tractability, much simpler models have been introduced (Durstewitz, 2003, 2009; Fourcaud-Trocmé et al., 2003; Izhikevich, 2004; Brette and Gerstner, 2005) as a phenomenological description of neuronal activity, often with a focus more on the dynamical mechanisms underlying spiking behavior. The leaky integrate-and-fire neuron (LIF; Lapicque, 1907; Hill, 1936) presumably presents the simplest spiking neuron model of this kind. In order to model the upswing of an action potential more faithfully, various extensions to the LIF neuron were proposed, like a quadratic function (Ermentrout and Kopell, 1986; Izhikevich, 2003) or an exponential term (Fourcaud-Trocmé et al., 2003; Brette and Gerstner, 2005) which captures the spike initiation phase. It was also shown that a second dynamical variable may be mandatory to capture certain neuronal features like adaptation (Izhikevich, 2003; Richardson et al., 2003). Models like the adaptive exponential integrate-and-fire model (AdEx; Brette and Gerstner, 2005) or the Izhikevich (2003) model can qualitatively reproduce a large number of spiking patterns observed in real neurons (Izhikevich, 2003, 2004; Naud et al., 2008; Durstewitz, 2009). In addition to the qualitative reproduction of real spike train features, a few studies also dealt with systematic quantitative fitting of model parameters on the basis of electrophysiological recordings (Jolivet et al., 2006, 2008; Clopath et al., 2007; Badel et al., 2008a,b; Naud et al., 2008; Gerstner and Naud, 2009). Remarkably, these simple neuron models, usually trained on *in vivo*-like fluctuating-current inputs, can often predict spike times from *in vitro* recordings with high precision.

Here, we suggest an alternative approach that rests mainly on firing rate information and simply uses standard *f*–*I* curves (firing rate over step current) for fitting model parameters. Such curves are routinely obtained by *in vitro* electrophysiologists and are widely available in public data bases for many different cell types. They cover a broad range of mean-input currents and output spike rates a neuron may traverse *in vivo*. To allow for a very fast and efficient fitting procedure, we derive an approximation to the AdEx model that results in closed-form expressions for transient and stationary firing rates. Spike-time prediction performance of the model is then tested, however, on different spike trains obtained from recordings with “*in vivo*-like” fluctuating-current injections. Model performance is evaluated on a large variety of experimentally recorded neocortical cell types, and is compared to an even simpler adaptive LIF neuron as well as to the full AdEx. Based on this large pool of experimental data, also some of the major empirical factors constraining the model fitting process are exposed. The result is a single-cell modeling and parameter fitting framework that allows to efficiently build up cell models for large pools of physiologically characterized cell types in relatively short time. Potential shortcomings and future extensions of our approach are discussed.

## 2 Materials and Methods

### 2.1 Training set data required for model fitting

The training set for the parameter tuning consists of onset and steady-state *f*–*I* curves as well as of the sub-rheobase *I*–*V* curve. The onset firing rate *f_O_*(*I*) reflects the initial response to a step-like current (*I*) stimulus (Benda and Herz, 2003) and is defined as the inverse of the first interspike interval (*ISI*).

(1) $$f_{O}\left(I\right)=\frac{1}{t_{O}^{ISI}\left(I\right)}.$$

However, sometimes initial spike doublets or triplets may be observed, partly reflecting the fact that neurons *in vitro* (in contrast to *in vivo*) usually reside at a lower membrane voltage at which many inward currents are in a recovered state, resulting in higher excitability initially when a current is first injected. To deal with this, the firing frequency adaptation curve is fitted by an exponential decay with an effective time constant τ_eff_ (Madison and Nicoll, 1984; Edman et al., 1987; Stocker et al., 1999; Benda and Herz, 2003), and the onset firing rate is determined by evaluating this function at the time of the first spike (a procedure which also results in a more stable estimate). The behavior of the adapted cell, on the other hand, is given by the steady-state firing rate *f*_∞_(*I*) defined as the inverse of the average interspike interval when the cell has reached a reasonably stationary level (i.e., an approximately constant firing rate).

(2) $$f_{\infty }\left(I\right)=\frac{1}{\left\langle t_{\infty }^{ISI}\left(I\right)\right\rangle }.$$

In practice, interspike intervals are averaged over an interval of 5 s after a transient of 10–15 s. Finally, the subthreshold behavior of the cell is assessed from those trials where the input currents are below the rheobase, i.e., do not cause spiking. The *I*–*V* curve was constructed by relating these input currents to the steady-state voltage response of the cell. For currents far below the rheobase, *I*(*V*) is often almost perfectly linear for rodent prefrontal cortex neurons (present observations).

### 2.2 Neuron models

We first briefly review the basic AdEx model in order to derive subsequently an approximation to it that allows for setting up closed-form expressions for the onset and steady-state *f*–*I* curves.

#### 2.2.1 The AdEx model

The AdEx model is a two-dimensional model that mathematically describes the evolution of the membrane potential *V*(*t*) and an adaptation current *w*(*t*). It is an extension of the exponential integrate-and-fire neuron (first developed in Fourcaud-Trocmé et al., 2003) and defined by the following system of non-linear ordinary differential equations (Brette and Gerstner, 2005; Naud et al., 2008):

(3) $$C\cdot \frac{dV}{dt}=-g_{L}\cdot \left(V-E_{L}\right)+g_{L}\cdot \Delta_{T}\cdot e^{\left(\frac{V-V_{T}}{\Delta_{T}}\right)}+I-w$$

$$\begin{array}{ll} \tau_{w}\cdot \frac{dw}{dt}=a\cdot \left(V-E_{L}\right)-w\\ \text{if}\;V>V_{\text{up}}\;\text{then}\;V\to V_{r}\;\text{and}\;w\to w_{r}=w+b & \text{(4)} \end{array}$$

The first equation of the AdEx is an extension of the LIF neuron that models the upswing of an action potential by an exponential function (Fourcaud-Trocmé et al., 2003). Whenever the membrane potential approaches the threshold *V_T_*, the exponential term causes a very rapid increase of the voltage (note that *V_T_* is not a strict spiking threshold, however, as in the classical LIF model, but just a parameter that determines where the exponential is “centered” on the V-axis). The downswing is replaced by a reset condition. In the second equation, the parameter *a* determines the subthreshold adaptation and *b* covers spike-triggered adaptation. Despite the simplicity of this two equation model with just a handful of parameters, it can reproduce a wide range of physiological firing patterns like tonic spiking, adaptation, initial or regular bursting, to name but a few (Naud et al., 2008). Further details of the AdEx model are described in Brette and Gerstner (2005) and Naud et al. (2008).

The nullclines of the ordinary differential equation (ODE) system of the AdEx provide insights into its dynamics. They are given by

(5) $$\frac{dV}{dt}=0\Rightarrow w_{V}=-g_{L}\cdot \left(V-E_{L}\right)+g_{L}\cdot \Delta_{T}\cdot e^{\left(\frac{V-V_{T}}{\Delta_{T}}\right)}+I$$

(6) $$\frac{dw}{dt}=0\Rightarrow w_{w}=a\cdot \left(V-E_{L}\right).$$

There are a maximum of two fixed points (associated with the state of resting) when *I* is small and zero fixed points (associated with a state of repetitive spiking) when *I* is sufficiently large. The transition from resting to spiking can occur through different types of bifurcation depending on parameter settings. For a saddle-node bifurcation, the Jacobian matrix *J* of the equations (3 and 4) has two real eigenvalues, one of them being equal to zero. This leads to the following condition at the bifurcation point *V*_0_.

(7) $$det\left(J\right)=0$$

(8) $$\Rightarrow V_{0}=V_{T}+\Delta_{T}\cdot ln\left(1+\frac{a}{g_{L}}\right).$$

Together with equations (5 and 6), this expression can be used to calculate the rheobase *I*_SN,0_.

(9) $$w_{V}\left(V_{0}\right)=w_{w}\left(V_{0}\right)$$

(10) $$\Rightarrow I_{\text{SN},0}=\left(a+g_{L}\right)\cdot \left[V_{T}-E_{L}-\Delta_{T}+\Delta_{T}\cdot ln\left(1+\frac{a}{g_{L}}\right)\right].$$

When *a* = 0, one can show that the transition from the resting state to repetitive spiking occurs always via a saddle-node bifurcation and the last intersection point (and with that the onset of the *f*–*I* curve) is determined by *V*_0_ = *V_T_* (Touboul, 2008; Touboul and Brette, 2008).

#### 2.2.2 An approximation to the AdEx model

The exponential term in the AdEx model renders an analytical solution of the differential equations impossible. Neither the membrane potential nor the *f*–*I* curves can be derived analytically. The fundamental issue is the lack of knowledge about the steady-state trajectory in the phase plane. One approach to solve the problem is to use an approximation to the AdEx based on the idea of separation of time scales: Under the assumption that the evolution of the *w*-variable is much slower than the evolution of the membrane potential $V\left(\frac{\tau_{m}}{\tau_{w}}<<1\right)$, the trajectory in the phase plane (Gerstner and Kistler, 2002; Naud et al., 2008)

- is nearly horizontal if it is far away from the V-nullcline *w_V_*,
- follows the left branch of that nullcline at a vertical distance *D*(*V*) as soon as it approaches the V-nullcline *w_V_*.

A slow adaptation is a reasonable assumption for many real neurons since the membrane time constant is often one to two orders of magnitude lower than the spike-rate adaptation time constant (Benda and Herz, 2003; Thomson and Lamy, 2007).

To obtain the steady-state firing rate without computing the whole transient dynamics, we require *w*(*t*) to be constant in time

(11) $$\frac{dw}{dt}=\frac{dw}{dV}\frac{dV}{dt}=0$$

*except* within a well-defined vicinity of the left branch of the V-nullcline *w_V_*. As *dV*/*dt* ≠ 0 away from *w_V_*, it follows that *dw*/*dV* = 0 in this case. Hence, the trajectory in the (*V,w*)-phase plane is exactly horizontal. In the second regime characterized by *dw*/*dt* ≠ 0, the trajectory has to follow a curve defined by a vertical distance *D*(*V*) up to its minimum at *V* = *V_T_*. Hence, *w*(*V*) is defined piecewise and we obtain a differential equation for the membrane potential *V* that can be solved section by section. Furthermore, the reset value *w_r_* and thus the value *w*(*V*_up_) for the onset and the steady state are known from the start.

To calculate *w* in the vicinity of *w_V_*, the vertical distance *D*(*V*) has to be specified. Following Gerstner and Kistler (2002) and Naud et al. (2008), we make the ansatz

(12) $$w_{\text{traj}}=w_{V}-\frac{1}{\tau_{w}}\cdot D\left(V\right).$$

Inserting (12) into equation (4) and using the additional assumption *a* = 0

(13) $$\frac{dw_{\text{traj}}}{dt}=-\frac{w_{\text{traj}}}{\tau_{w}}=-\frac{w_{V}}{\tau_{w}}+\frac{D\left(V\right)}{\tau_{w}^{2}}.$$

Using (3) and differentiating *w*_traj_ [equations (12) and (5)] with respect to V, *dw*_traj_/*dt* can also be obtained as

(14) $$\frac{dw_{\text{traj}}}{dt}=\frac{dw_{\text{traj}}}{dV}\frac{dV}{dt}=\left(\;-\frac{1}{\tau_{m}\tau_{w}}+\frac{e^{\left(\frac{V-V_{T}}{\Delta_{T}}\right)}}{\tau_{m}\tau_{w}}-\frac{D^{\prime }\left(V\right)}{C\tau_{w}^{2}}\;\right)D\left(V\right).$$

where τ*_m_* = *C*/*g_L_* is the membrane time constant. In obtaining this expression we also used the relationship *C*·*dV*/*dt* = *D*(*V*)/τ*w* that follows from inserting (5) into (12). Setting (13) = (14) and solving for *D*(*V*) one obtains the approximation

(15) $$D\left(V\right)\approx \frac{C}{g_{L}}\cdot \left(\;I+g_{L}\cdot \Delta_{T}\cdot e^{\left(\frac{V-V_{T}}{\Delta_{T}}\right)}-g_{L}\cdot \left(V-E_{L}\right)\;\right)\;=\tau_{m}\cdot w_{V}$$

under the assumptions that $\tau_{w}^{-2}$ is sufficiently small such that terms containing $\tau_{w}^{-2}$ can be neglected, and ${\left(\tau_{\text{w}}\cdot \tau_{m}\right)}^{-1}\cdot e^{\frac{V-V_{T}}{\Delta_{T}}}\approx 0.$ This latter assumption is valid provided that (τ*_w_*·τ*_m_*) is sufficiently large compared to the exponential term which is ≤1 for all values *V* ≤ *V_T_*. Inserting (15) into (12), *w*_traj_ is approximately given by

(16) $$w_{\text{traj}}\approx \left(1-\frac{\tau_{m}}{\tau_{w}}\right)\cdot w_{V}$$

and we can therefore write

(17) $$\frac{dw}{dt}=\frac{dw}{dV}\frac{dV}{dt}=\left(1-\frac{\tau_{m}}{\tau_{w}}\right)\frac{dw_{V}}{dV}\frac{dV}{dt}.$$

Defining two functions *e_l_* and *e_r_* by

(18) $$e_{l}=w_{V}-\frac{D\left(V\right)}{\tau_{w}}=\left(1-\frac{\tau_{m}}{\tau_{w}}\right)w_{V}$$

(19) $$e_{r}=w_{V}+\frac{D\left(V\right)}{\tau_{w}}=\left(1+\frac{\tau_{m}}{\tau_{w}}\right)w_{V},$$

the second regime is confined within the band [*e_l_*, *e_r_*] for *V* ≤ *V_T_*.

**Figure 1:**
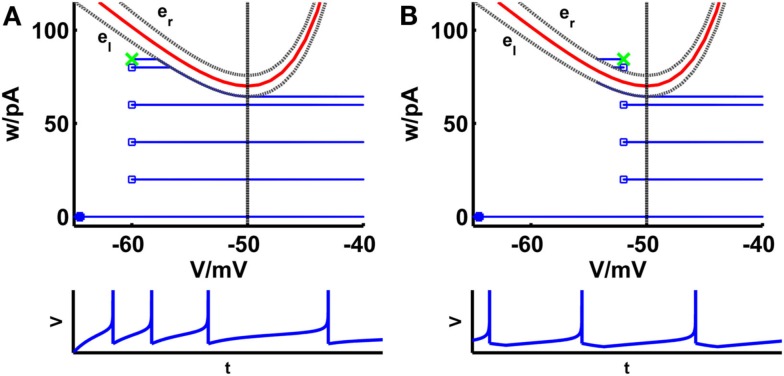
**Comparison of phase planes of the simpAdEx for sharp (A) and broad (B) reset**. If the steady-state reset point [(*V_r_*, *w_r_*); green cross] is below the V-nullcline (red curve) the spiking pattern corresponds to a sharp reset (no undershoot), otherwise it is broad (associated with an after-hyperpolarizing current or undershoot). The trajectory is given in blue with the blue filled square representing the initial point and the open blue squares indicating the reset points. The lower panels show the voltage traces corresponding to these trajectories. The dashed vertical line marks the threshold V_T_ beyond which the trajectory runs strictly parallel to the abscissa. The two gray dashed lines present the functions e_l_ and e_r_ described in the text. Note that the distances of the envelopes to the V-nullcline have been enlarged for clarity.

*Simplified AdEx model:* Summing up the results from the previous section, the simplified AdEx model (simpAdEx) is defined as follows:

(20) $$C\cdot \frac{dV}{dt}=-g_{L}\cdot \left(V-E_{L}\right)+g_{L}\cdot \Delta_{T}\cdot e^{\left(\frac{V-V_{T}}{\Delta_{T}}\right)}+s\cdot I-w=w_{V}-w$$

$$\begin{array}{ll} \frac{dw}{dt}=\left\{\;\;\begin{array}{cc} 0 & \text{for}\;D<\tau_{w}\cdot \left|w-w_{V}\right|\\ \Theta \left(V_{T}-V\right)\cdot \left[1-\frac{\tau_{m}}{\tau_{w}}\right]\frac{dw_{V}}{dV}\frac{dV}{dt} & \;\text{otherwise} \end{array}\right. & \text{(21)}\\ \text{if}\;V>V_{\text{up}}\;\text{then}\;V\to V_{r}\;\text{and}\;w\to w_{r}=w+b\\ \text{if}\;w=\left(1+\frac{\tau_{m}}{\tau_{w}}\right)w_{V}\;\text{then}\;w\to \left(1-\frac{\tau_{m}}{\tau_{w}}\right)w_{V} \end{array}$$

where Θ denotes the Heaviside function and *s* corresponds to a scaling factor set to 1 for the fitting process (see Section 3.2 for further explanation). The first if-condition describes the downswing. It is equivalent to the reset defined in the AdEx. The second if-condition defines a vertical jump from the envelope *e_r_* to *e_l_* as soon as the trajectory reaches the curve *e_r_*. This constraint is necessary because of a singularity in the integral that would appear whenever the horizontal trajectory crosses the V-nullcline. Thus, the trajectory is horizontal unless in close proximity to the left branch of the V-nullcline, where it follows the branch at a vertical distance defined by *D*. We can now distinguish between two cases in the steady state:

1. The steady-state reset value *w_r_* is below the V-nullcine *w_V_* and the trajectory approaches the left branch from the left. As soon as the horizontal trajectory crosses the curve *e_l_*, it follows the curve up to the point *V* = *V_T_*. From there on it stays horizontal at *w_V_*(*V_T_*) − *D*(*V_T_*)/τ*_w_*. The corresponding voltage trace shows a sharp reset (Figure 1A).
2. The steady-state reset value *w_r_* is above the V-nullcline *w_V_* and the trajectory approaches the left branch from the right. As soon as the horizontal trajectory crosses the curve *e_r_*, it jumps vertically to the curve *e_l_*, where it follows *e_l_* up to the point *V* = *V_T_*. From there on it stays horizontal at *w_V_*(*V_T_*) − *D*(*V_T_*)/τ*_w_*. The corresponding voltage trace shows a broad reset (Figure 1B).

**Figure 2:**
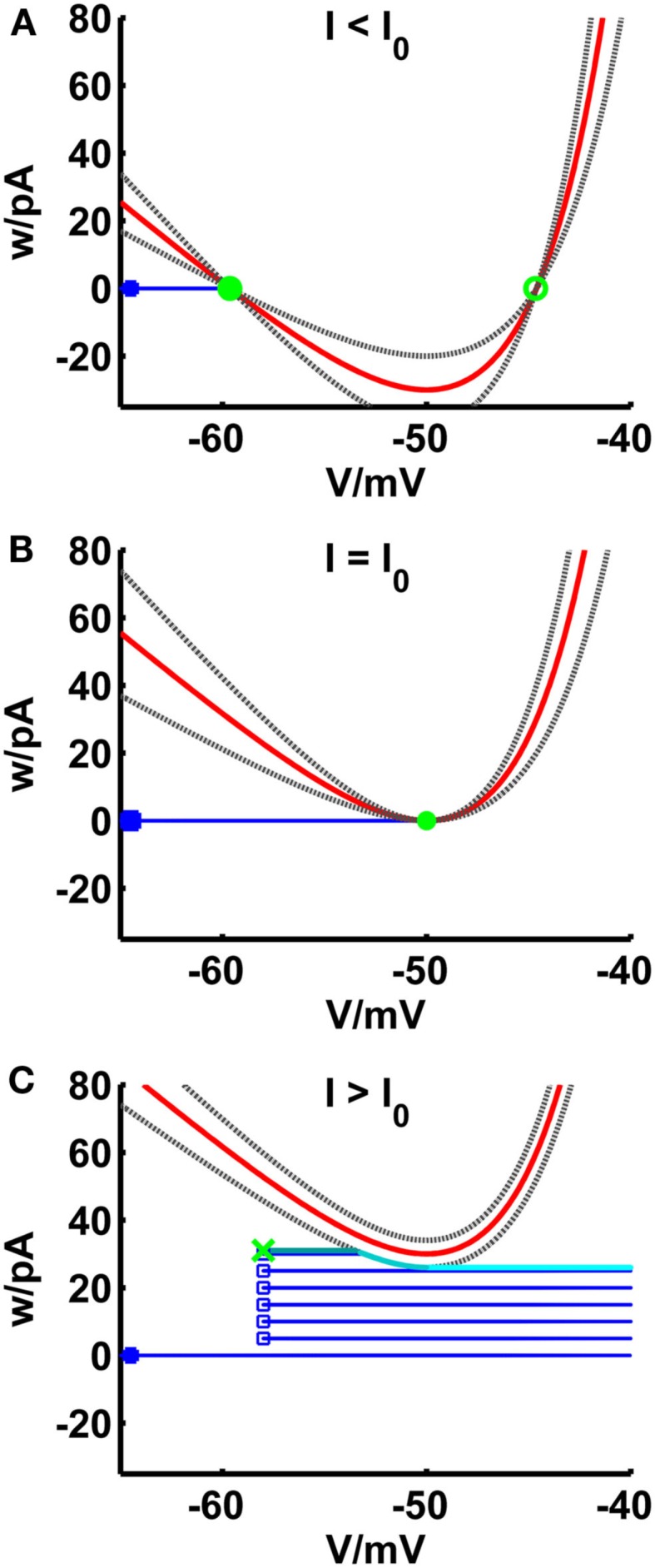
**Comparison of phase planes of the simpAdEx for *I* less than (A), equal (B) and greater than (C) the rheobase**. The V-nullcline (in red) and the curves e_l_ and e_r_ (gray dashed lines) are shifted upwards with increasing input current. For I less than or equal the rheobase, the three curves have a maximum of two shared intersection points (one stable node given by the filled green circle and one unstable node represented by an open green circle) which coincide at the saddle point *V* = *V_T_*. The trajectory is given in blue with the blue filled square representing the initial point and the open blue squares indicating the reset points. The steady-state trajectory starts at the green cross and passes through three sections: It runs horizontal up to the intersection point with the envelope *e_l_* (dark cyan line) where it follows *e_l_* up to its minimum at *V_T_* (cyan line). The third part of the trajectory is again horizontal (light cyan line). Note that the distances of the curves *e_l_* and *e_r_* to the V-nullcline have been enlarged for clarity.

The fixed points of the system are the intersection points of the V-nullcline with the horizontal *w* = 0. Figure 2 shows the behavior of the V-nullcline as well as the functions *e_l_* and *e_r_* with increasing input current *I* in the phase plane. The curves are shifted upwards and for *I* less than or equal to the rheobase, they have at least one intersection point. In equations (13 and 14), the subthreshold adaptation defined by *a* was set to zero such that the intersection points of *e_l_* and *e_r_* coincide with the fixed points of the system. Hence, it is ensured that the sub-rheobase *I*–*V* curve is consistently defined by equations (20 and 21).

(22) $$I\left(V\right)=g_{L}\cdot \left(V-E_{L}\right)-g_{L}\cdot \Delta_{T}\cdot e^{\frac{V-V_{T}}{\Delta_{T}}}\;\;\forall \;V\leq V_{T}.$$

The system has a maximum of two fixed points that coalesce and finally disappear via a saddle-node bifurcation at *V_T_* since *a* = 0 [cf. equation (10)].

Due to the piecewise defined *w*(*V*), we can now derive a closed-form expression for the onset $t_{O}^{ISI}\left(I\right)$ and steady-state interspike interval $t_{\infty }^{ISI}\left(I\right)$. Based on equation (20) and using separation of variables, the solution is given in terms of integrals of the general form:

(23) $$t^{ISI}=\int_{V\left(t_{a}\right)}^{V\left(t_{e}\right)}\;\frac{C\cdot dV}{w_{V}\left(V\right)-w\left(V\right)}.$$

Specifically, the steady-state interspike interval $t_{\infty }^{ISI}\left(I\right)$ can be computed as the sum of three integrals over the three different regimes in the phase plane (Figure 2C):

(24) $$t_{\infty ,1}^{ISI}=\int_{V_{r}}^{V_{s}}\;\frac{C\cdot dV}{w_{V}\left(V\right)-w_{r}}$$

(25) $$t_{\infty ,2}^{ISI}=\int_{V_{s}}^{V_{T}}\;\frac{C\cdot \tau_{w}\cdot dV}{D\left(V\right)}$$

$$\begin{array}{rcl} t_{\infty ,3}^{ISI} & =\int_{V_{T}}^{V_{\text{up}}}\;\frac{C\cdot dV}{w_{V}\left(V\right)-w_{r}+b}\\ f_{\infty } & ={\left[t_{\infty ,1}^{ISI}+t_{\infty ,2}^{ISI}+t_{\infty ,3}^{ISI}\right]}^{-1}. & \text{(26)} \end{array}$$

where *V_s_* denotes the intersection point of the horizontal at *w_r_* with the curve *e_l_* when the steady-state reset point is below the V-nullcline, and with *e_r_* otherwise. This relationship holds only for *b* > 0, while for *b* = 0 the steady-state interspike interval is given by $t_{\infty }^{ISI}=t_{\infty ,1}^{ISI}$ with *V_s_* = *V*_up_. The onset *f*–*I* curve is given by

(27) $$f_{O}={\left[t_{O}^{ISI}\right]}^{-1}\;\text{with}\;t_{O}^{ISI}=\int_{V_{r}}^{V_{\text{up}}}\;\frac{C\cdot dV}{w_{V}\left(V\right)-b}.$$

Equation (27) is only valid for *b* ≤ *w_V_*(*V_T_*) − D(*V_T_*)/τ*_w_*. Otherwise $t_{O}^{ISI}$ has also to be split up into three integrals, as for the steady state, since the trajectory would reach the vicinity of the V-nullcline. Note that equations (24–27) still need to be solved numerically since analytical expressions for the integrals are not available. This, however, can be done through fast algorithms like the adaptive Lobatto quadrature (Matlab function “quadl”) which evaluates the integral only at a few discrete points. The resulting fitting procedure is about 10–100 times faster than explicitly simulating whole voltage traces up to where a steady-state in the firing rate is reached (see Table 2; simulating the full (*V,w*)-trajectory, however, is about as time-consuming as for the full AdEx). The mathematical derivations above were also checked numerically (i.e., by simulation of the full system).

**Figure 3:**
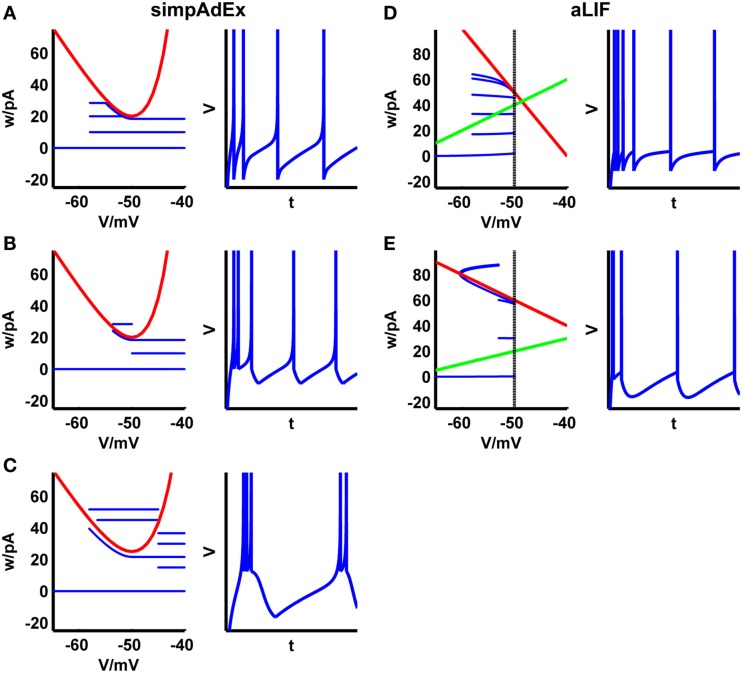
**Phase plane representations and corresponding spiking patterns upon constant current steps for the approximation to the AdEx (A–C) and the adaptive LIF neuron (D,E)**. Adaptation **(A,D)**, initial burst **(B,E)**, and regular bursting **(C)**. Red curve: V-nullcline, blue curve: trajectory. For the adaptive LIF model, the w-nullcline is shown in green and the dashed vertical marks the threshold *V_T_*.

Despite these simplifications, the simpAdEx can reproduce many of the spiking patterns observed in real neurons and as seen in the full AdEx, including tonic spiking, adaptation, initial bursting, or regular bursting (Figures 3A–C). In addition to the closed-form expressions for the onset and steady-state firing rate, one can also easily find similar expressions for the latency or the number of spikes in response to a step current.

#### 2.2.3 The adaptive LIF model

As an alternative to the simpAdEx model above, we also investigated an even simpler model which incorporates subthreshold adaptation (*a* ≠ 0) but allows for an analytical solution for the *f*–*I* curves, namely the adaptive LIF neuron defined by

(28) $$C\cdot \frac{dV}{dt}=-g_{L}\cdot \left(V-E_{L}\right)+I-w$$

$$\begin{array}{ll} \tau_{w}\cdot \frac{dw}{dt}=a\cdot \left(V-E_{L}\right)-w & \text{(29)}\\ \text{ if}\;V>V_{T}\;\text{then}\;V\to V_{r}\;\text{and}\;w\to w_{r}=w+b \end{array}$$

This model is identical to the AdEx except it lacks the exponential term describing the action potential upswing. For *b* = 0, it is well described and discussed in Izhikevitch (2001) and Richardson et al. (2003). The nullclines are given by

(30) $$\frac{dV}{dt}=0\Rightarrow w_{V}=-g_{L}\cdot \left(V-E_{L}\right)+I$$

(31) $$\frac{dw}{dt}=0\Rightarrow w_{w}=a\cdot \left(V-E_{L}\right).$$

The system has either no fixed points when the nullclines run parallel (*I* ≠ 0 and *a* = −*g_L_*), infinitely many fixed points when the nullclines lie on top of each other (*I* = 0 and *a* = −*g_L_*), or exactly one fixed point whose eigenvalues are given by

$$\begin{array}{ll} \lambda_{1,2}=\frac{1}{2}\left[-\left(\frac{1}{\tau_{m}}+\frac{1}{\tau_{w}}\right)\pm \sqrt{{\left(\frac{1}{\tau_{\text{m}}}+\frac{1}{\tau_{\text{w}}}\right)}^{2}-4\cdot \frac{a+g_{L}}{C\cdot \tau_{w}}}\right]\\ =\;\frac{1}{2}\left[-m\pm \sqrt{\Delta }\right], & \text{(32)} \end{array}$$

and which is stable for

(33) $$a\geq -g_{L}.$$

The transition to repetitive spiking can be obtained by adjusting the threshold parameter *V_T_* which defines the reset condition. Increasing *I* shifts the intersection point to the right. The rheobase is then defined by the intersection point at the threshold parameter *V_T_*: *I*_0_ = (*g_L_* + *a*)·(*V_T_* − *E_L_*). The system has three dynamical regimes depending on its eigenvalues λ_1_ and λ_2_, and the general solution is given by

(34) $$V\left(t\right)=\left\{\;\begin{array}{cc} C_{1}\cdot e^{\lambda_{1}\cdot t}+C_{2}\cdot e^{\lambda_{2}\cdot t}+\frac{s}{n} & \text{if}\;\Delta >0\\ \left(C_{1}+C_{2}\cdot t\right)\cdot e^{-\frac{m}{2}\cdot t}+\frac{s}{n} & \text{if}\;\Delta =0\\ e^{-\frac{m}{2}\cdot t}\cdot [C_{1}\cdot cos\left(\beta t\right)\;+ & \text{if}\;\Delta <0\;\text{with }\beta =\frac{\sqrt{-\Delta }}{2}\\ C_{2}\cdot sin(\beta t)]+\frac{s}{n} \end{array}\right.$$

where *s* = (τ*_w_*·*C*)^−1^·[*I* + (*a* + *g_L_*)·*E_L_*], $n=a+g_{L}∕\tau_{w}\cdot C$, and the parameters *C*_1_ and *C*_2_ have to be determined from boundary conditions. For the onset and steady state, these boundary conditions can be written as

$$\begin{array}{ll} \left(V\left(0\right),\dot{V}\left(0\right)\right)\\ =\left\{\;\begin{array}{cc} \left(E_{L},\frac{I}{C}\right) & \text{for}\;\text{the}\;\text{latency}\;\left(\text{lat}\right)\\ \left(V_{r},{\dot{\text{V}}}_{\text{lat}}\left(t_{l}\right)-\frac{g_{L}}{C}\left(V_{r}-V_{T}\right)-\frac{b}{C}\right) & \text{for}\;\text{the}\;\text{initial}\;\text{state}\\ \left(V_{r},\frac{1}{C}\left[-g_{L}\cdot \left(V_{r}-E_{L}\right)+I-w_{r}\right]\right) & \text{for}\;\text{the}\;\text{steady}\;\text{state} \end{array}\right. & \text{(35)} \end{array}$$

In the following, we derive analytical solutions for the onset and steady-state firing rates for the first regime defined by Δ > 0 as an example. Analog expressions for the cases Δ = 0 and Δ < 0, respectively, can be directly deduced. In order to calculate the onset firing rate, we first have to consider the latency defined as the time delay up to the first spiking time. By using the boundary conditions for the latency and solving the resulting system of equations, we obtain the following equation for the membrane potential *V*_lat_(*t*):

(36) $$V_{\text{lat}}\left(t\right)=\left[\frac{I}{C}-E_{L}^{*}\cdot \lambda_{2}\right]\cdot \frac{e^{\lambda_{1}\cdot t}-e^{\lambda_{2}\cdot t}}{\lambda_{1}-\lambda_{2}}+E_{L}^{*}\cdot e^{\lambda_{2}\cdot t}+\frac{s}{n}.$$

with $E_{L}^{*}=E_{L}-s∕n$. The latency time *t_l_* can be calculated by taking *V*_lat_(*t_l_*) = *V_T_*:

(37) $$V_{\text{T}}^{*}=\left[\frac{I}{C}-E_{L}^{*}\cdot \lambda_{2}\right]\cdot \frac{e^{\lambda_{1}\cdot t_{l}}-e^{\lambda_{2}\cdot t_{l}}}{\lambda_{1}-\lambda_{2}}+E_{L}^{*}\cdot e^{\lambda_{2}\cdot t_{l}}.$$

where $V_{\text{T}}^{*}$ is given by $V_{\text{T}}^{*}=V_{T}-s∕n.$ On the basis of the solution (36), ${\dot{\text{V}}}_{\text{lat}}\left(t_{l}\right)$ can be derived. A closed-form expression for the onset as well as the steady-state interspike interval (in the following denoted by *t^ISI^*, with $t^{ISI}=t_{\infty }^{ISI}$ and $t^{ISI}=t_{O}^{ISI},$ respectively), can be derived with the boundary condition *V*(0) = *V_r_* and *V*(*t^ISI^*) = *V_T_*:

(38) $$V_{\text{T}}^{*}=L\left(V_{r},\dot{V}\left(0\right)\right)\cdot \left[e^{\lambda_{1}\cdot t^{ISI}}-e^{\lambda_{2}\cdot t^{ISI}}\right]+V_{r}^{*}\cdot e^{\lambda_{2}\cdot t^{ISI}}$$

where $V_{r}^{*}=V_{r}-s∕n,\;\;V_{\text{T}}^{*}=V_{T}-s∕n\;\;\text{and}\;L\left(V_{r},\dot{V}\left(0\right)\right)$ denotes *L_O_* for the initial regime and *L*_∞_ for the steady state, respectively. In the case of the initial state, *L_O_* is given by

(39) $$L_{O}=\frac{1}{\lambda_{1}-\lambda_{2}}\cdot \left[{\dot{\text{V}}}_{\text{lat}}\left(t_{l}\right)-\frac{g_{L}}{C}\left(V_{r}-V_{T}\right)-\frac{b}{C}-\lambda_{2}\cdot V_{r}^{*}\right].$$

The calculation of the steady-state firing rate necessitates a further condition:

(40) $$w\left(t_{\infty }^{ISI}\right)=w_{r}-b=-g_{L}\left(V_{T}-E_{L}\right)+I-C\cdot \frac{dV}{dt}\left|t_{\infty }^{ISI}\right..$$

Using this, *L*_∞_ is a linear function of *w_r_*:

(41) $$L_{\infty }=\frac{1}{\lambda_{1}-\lambda_{2}}\cdot \left[\frac{1}{C}\left(w_{V}\left(V_{r}\right)-w_{r}\right)-\lambda_{2}\cdot V_{r}^{*}\right].$$

*w_r_* in turn is uniquely given by (40) with

(42) $$\frac{dV}{dt}\left|t_{\infty }^{ISI}\right.=L_{\infty }\cdot \left[\lambda_{1}\cdot e^{\lambda_{1}\cdot t_{\infty }^{ISI}}-\lambda_{2}\cdot e^{\lambda_{2}\cdot t_{\infty }^{ISI}}\right]+\lambda_{2}\cdot V_{r}^{*}\cdot e^{\lambda_{2}\cdot t_{\infty }^{ISI}}.$$

Equations (38) and (39) determine the initial interspike interval $t_{O}^{ISI}$ and equations (38), (41), and (42) can be combined into one equation which gives a closed-form expression for the steady-state interspike interval. The onset and the steady-state firing rates are then directly given by the inverse of these expressions.

The model cannot reproduce as many spiking patterns as the AdEx, but shows notable features like tonic spiking, adaptation, transient spiking, or delayed acceleration (Figures 3D,E). Based on this formalism, piecewise defined linear models may increase the variety of spiking patterns while remaining mathematically tractable.

### 2.3 Model fitting method

*Estimating initial parameter values:* Most of the parameters of the model are subject to a fitting procedure. The only exceptions are the capacitance *C* and the upper limit of the membrane potential *V*_up_ which are set by explicit constraints. *C* is fixed by the relation *C* = *g_L_*·τ*_m_*, where the membrane time constant τ*_m_* is directly obtained from subthreshold recordings. Specifically, τ*_m_* is extracted from exponential fits to the initial part (~50–100 ms) of voltage traces after applying small hyper- or depolarizing current steps. A single exponential term fitted the decay (rise) reasonably well for our recordings. *V*_up_, on the other hand, does not significantly affect the model dynamics and was coupled to the fitted slope factor Δ*_T_* through *V*_up_ = 10·Δ*_T_* − 40 to avoid numerical problems.

Where possible, initial values were taken directly from the data: *E_L_* and *g_L_* are extracted as the offset and slope, respectively, of the linear fit to the sub-rheobase *I*–*V* curve in its approximately linear range. Furthermore, an estimate of the threshold *V_T_* can be derived from the *I*–*V* curve of the simpAdEx model. As the cell does not spike and thus *w* = 0, the current *I* that results in a given voltage *V* can be calculated by searching for the zero crossings of the V-nullcline *w_v_*. From equation (22), it follows that

(43) $$I_{0}=g_{L}\cdot \left(V_{T}-E_{L}\right)-g_{L}\cdot \Delta_{T}.$$

Equation (43) suggests that a reasonable estimate for *V_T_* is given by the voltage at the rheobase current *I*_0_. A log-function is fitted to the empirical *f*–*I* curve for estimating the rheobase, and the corresponding value for *V* is used as initial estimate for *V_T_*.

The remaining parameters Δ*_T_*, τ*_w_*, *V_r_*, and *b* do not have a clearly defined physiological equivalent (although Δ*_T_* may be mainly related to the fast Na^+^ channel activation). Based on experience, the slope factor Δ*_T_* is usually around 1–3 mV (Fourcaud-Trocmé et al., 2003; Clopath et al., 2007; Badel et al., 2008a; Naud et al., 2008), so 2 mV was used as an initial estimate. A rough estimate of the reset value *V_r_* was taken directly from the voltage traces upon step currents. For *b*, we defined a lower bound by the inverse of the time constant *τ*_eff_ of the adaptation, as small values of *b* lead to a very slow adaptation.

*The fitting procedure:* All software used for parameter tuning and model validation was written in Matlab and C and will be made publicly available at www.bccn-heidelberg-mannheim.de. Since closed-form expressions are available for the final interspike intervals, it is no longer necessary to numerically integrate the full two-dimensional (*V,w*)-trajectory up to the point where a steady state in spiking activity has been reached. In addition, equations (24–27) allow for faster numerical schemes that evaluate the integrals only at specific points, like adaptive Lobatto quadrature (Press et al., 2007) as provided by the built-in Matlab function “quadl.” Our fitting algorithm has three consecutive steps. During the first and the second step, the parameters are first roughly tuned by fitting only three points of the *I*–*V* curve and the *f*–*I* curves, respectively, in order to refine the initial estimates. More precisely, uniformly distributed pseudorandom numbers on an interval defined by ±20% of the initial parameter estimates are used to fit two data points near the rheobase of the *I*–*V* curve and one point far away from it, in order to capture roughly the subthreshold behavior and the onset of the *f*–*I* curves. During this optimization step, only the parameters *g_L_*, *E_L_*, Δ*_T_*, and *V_T_* are tuned. The membrane capacitance *C* is then recalculated by *C* = τ*_m_*·*g_L_* since τ*_m_* is assumed to be fixed. Next, the slopes of the simulated onset and steady-state *f*–*I* curves are adjusted simultaneously by using three data points (the rheobase, one close to the onset and the last defined point) of the real *f*–*I* curves. In this process, the parameters *b*, τ*_w_*, and *V_r_* are optimized and the other parameters are held fixed because the slope is mainly determined by these three. Since we do not have initial values for the first two parameters, several combinations of systematically chosen initial values for τ*_w_* and *b* are tested. Subsequently, the fitting errors are compared and the pair (τ*_w_*, *b*) corresponding to the smallest error is taken for the final fitting step. In the final step, all data points of the three curves are fitted simultaneously, allowing all parameters except Δ*_T_*, *V*_up_, *g_L_*, and *C* to be optimized. We emphasize that this whole fitting procedure is completely automatized and does not require any parameter setting/exploration or pre-inspection of data by the user: It requires nothing more than provision of the training data, from which the routine extracts initial estimates as described above, and then automatically cycles through all the steps above until a fixed convergence criterion is reached.

The optimization function is given by

$$\begin{array}{ll} q=w_{1}\cdot \sum {\left(f_{\infty ,\text{target}}-f_{\infty ,\text{model}}\right)}^{2}\\ +w_{2}\cdot \sum {\left(f_{\text{O},\text{target}}-f_{\text{O},\text{model}}\right)}^{2}+w_{3}\cdot \sum {\left(I_{\text{target}}-I_{\text{model}}\right)}^{2} & \text{(44)} \end{array}$$

The weights *w* = (*w*_1_,*w*_2_,*w*_3_) may be chosen to balance the relative importance of the three contributions. We set *w* = (5,1,4) as from our observations the steady-state *f*–*I* curve appeared to be most important for the spiking behavior. The whole procedure is repeated at least five times starting from different initial estimates. In all cases this took less than 15 min on a single 2.4 GHz Intel(R) Xeon(R) CPU E5620 (for comparison with the full AdEx, see Table 2). The optimized parameter configuration with the lowest overall fitting error is then used to predict the spike times in test sets consisting of voltage traces upon fluctuating-current input. Generally, we found that different initial estimates resulted in similar parameter configurations.

### 2.4 Performance measure

We used two previously introduced performance measures to evaluate the prediction quality of our model more formally: The coincidence rate Γ and the Victor–Purpura measure *D^VP^*(*q*). The coincidence rate (Kistler et al., 1997; Gerstner and Kistler, 2002; Jolivet et al., 2004, 2008) basically describes the percentage of correctly predicted spike times with precision Δ, taking stochastic coincidences into account

(45) $$\Gamma =\frac{N_{\text{coinc}}-\left\langle N_{\text{coinc}}\right\rangle }{0.5\left(N_{\text{model}}+N_{\text{data}}\right)}\frac{1}{N_{\text{norm}}}.$$

where *N*_coinc_ is the number of coincidences within ±Δ, and 〈*N*_coinc_〉 = 2νΔ*N*_data_ is the expected number of coincidences generated by a homogeneous Poisson process with rate ν. The variables *N*_model_ and *N*_data_ denote the number of spikes in the spike trains generated by the model and the real neuron, respectively, and *N*_norm_ = 1 − 2νΔ is a normalization factor (Kistler et al., 1997; Gerstner and Kistler, 2002; Jolivet et al., 2004, 2008).

The Victor–Purpura measure (Victor and Purpura, 1996) is a metric based on spike times or interspike intervals and can be understood as a cost function that specifies how much effort is needed to transfer one spike train into the other. The measure depends on a cost parameter *q* that describes the relative sensitivity of the metric to precise timing of spikes. The algorithm to calculate the measure is best described by the elementary steps that are allowed: adding or deleting a spike has the cost of 1 and shifting a spike by the amount Δ*t* is equivalent to the cost of *q*|Δ*t*|. Following Kreiman et al. (2000), we normalize the Victor–Purpura metric by the total number of spikes in both spike trains to ensure that the value is always between 0 and 1. To facilitate comparability with the coincidence rate, we rearrange the measure such that a value of 0 is equivalent to no similarity and 1 indicates the best scenario:

(46) $$VP\left(q\right)=1-\frac{D^{VP}\left(q\right)}{N_{\text{model}}+N_{\text{data}}}.$$

Both these measures were also used to determine the intrinsic reliability of experimentally recorded cells, i.e., the coincidence of spikes between different identical repetitions of the same stimulus injected into the same neuron. We report the intrinsic reliability as the average over all pairs of repetitions.

### 2.5 Experimental preparation and electrophysiological recordings

Coronal cortical slices (250–300 μm) containing the prelimbic/intralimbic region of the medial PFC were prepared from the brains of 44–55 days old BL/6 mice and Sprague Dawley rats following decapitation, in accordance with German animal welfare laws and institutional regulations. The brains were rapidly dissected and brain slices were prepared in cold (4°C), oxygenated (carbogen, 95% O_2_–5% CO_2_) ACSF containing (in mM): 124 NaCl, 3 KCl, 1.8 MgSO_4_, 1.6 CaCl_2_, 10 Glucose, 1.25 NaH_2_PO_4_, and 26 NaHCO_3_. Slices were then transferred to a chamber containing ACSF at room temperature. Submerged slices in the recording chamber were continuously perfused with oxygenated ACSF. Neurons were identified based on their somatic morphology and the orientation of their dendrites (visualized using differential interference contrast microscopy). Pyramidal cells had triangular shaped somas and prominent apical dendrites (Mason et al., 1991; Schröder and Luhmann, 1997), bitufted cells elongated somas with one or two prominent, vertically orientated dendrites (Reyes et al., 1998; Rozov et al., 2001), and fast-spiking cells had round cell bodies with multipolar dendrites (Connors and Gutnick, 1990). Neuronal identity was further confirmed by their characteristic action potential firing patterns in response to stepped depolarization (Connors et al., 1982; Mason and Larkman, 1990; Chagnac-Amitai et al., 2004). Thick-walled borosilicate pipettes (6–8 MΩ tip resistance) were used for the whole cell patch-clamp recordings and were filled with (in mM): 105 K-gluconate, 30 KCl, 10 HEPES, 4 MgATP, 0.3 GTP, and 10 Phosphocreatine. Recordings were made using an Axoclamp 2B amplifier (Axon Instruments, Union City, CA, USA). Data was filtered at 2 kHz and digitized at 10–20 kHz with an ITC-16 (InstruTech, Port Washington, NY, USA) and analyzed offline using customized Matlab analysis routines (MathWorks, MA, USA). All recordings were performed at 33–36°C. Series resistance was not compensated in most of the recordings, and no adjustments to membrane potential were made. In all experiments 50 μM Picrotoxin (GABA_A_ receptor blocker), 50 μM DNQX (AMPA receptor antagonist), and 50 μM dl-2-amino-5-phosphonovaleric acid (NMDA receptor blocker) was added to the recording solution in order to minimize synaptic noise.

For the recording of *f*–*I* curves and sub-rheobase *I*–*V* curves, current steps from −200 up to 600 pA were applied for 25 s each. The depolarizing inputs were preceded by a brief hyperpolarizing current step of −50 pA for monitoring input resistance stability. Interleaved with these constant current protocols, fluctuating-current inputs were applied to the cell for 25 s if the mean current was greater than or equal the rheobase and 45 s otherwise in order to generate a reasonable number of spikes. The step- and fluctuating-current parts of the full recording protocol were repeated about 2–8 times in total (with each repeat identical; each part took about 4–6 min, separated by ~30 s), to account for physiological variability and to obtain estimates of cellular reliability. A total of ~100 prefrontal cortex cells were recorded using this type of protocol. Fluctuating currents were constructed from two Poisson spike trains mimicking 100 excitatory input neurons each firing at 10 Hz and 200 inhibitory neurons each at 20 Hz, respectively. The spike trains were filtered by an artificial synapse modeled by double exponential functions with the kinetics of AMPA, GABA_A_, and NMDA currents. The parameters for the synaptic kinetics (τ_on_ = [0.5, 1, 2.5] ms and τ_off_ [2, 6, 95] ms for AMPA, GABA_A_, and NMDA, respectively), and the non-linearity of the NMDA current (see Jahr and Stevens, 1990), were taken from Durstewitz (2003). The mean and SD of the total current input were controlled by changing synaptic weights, i.e., by the effect each spike has on the three different current components. Model parameters were fitted to the *f*–*I* and sub-rheobase *I*–*V* curves, and prediction performance was evaluated on the block of fluctuating current sets immediately following the respective training set.

## 3 Results

We investigated a large number of data sets (*N* ~ 100 recorded prefrontal cortex neurons) in order to characterize the potential of our simplified AdEx model (the AdEx model is originally developed in Brette and Gerstner, 2005). This was done by fitting parameters of the model on onset and steady-state *f*–*I* and sub-rheobase *I*–*V* curves, and subsequently evaluating the prediction performance on test sets consisting of *in vivo*-like fluctuating input currents (Destexhe et al., 2001, 2003).

The onset *f*–*I* curve *f_O_*(*I*) captures the initial response of the non-adapted cell, while the steady-state *f*–*I* curve *f*_∞_(*I*) reflects the behavior of the adapted cell for a given level *I* of mean input. The sub-rheobase *I*–*V* curve in addition captures subthreshold and passive response properties of the cell. This training set is easily obtained by standard current-clamp protocols with hyper- and depolarizing constant current steps (see Materials and Methods) and thus widely available (Benda and Herz, 2003). The fitting procedure is based on closed-form expressions for the *f*–*I* curves we had derived from an approximation to the AdEx model (see Materials and Methods), and therefore does not require to simulate the full underlying system of differential equations up to the point where a steady-state in spiking activity has been reached. It also allows for faster numerical schemes. In consequence, the resulting fitting scheme is about one to two orders of magnitude faster than methods that require numerical integration of the underlying system of differential equations (see Table 2 and below).

Fluctuating inputs mimicking synaptic bombardment (Destexhe and Paré, 1999; Destexhe et al., 2001, 2003) had been used previously for the purpose of model fitting (Jolivet et al., 2006, 2008; Clopath et al., 2007; Badel et al., 2008a,b; Naud et al., 2008; Gerstner and Naud, 2009). In our case, however, these traces will be used purely or mainly for checking the prediction performance of the model, i.e., either no information at all (Section 3.1) or only total spike count (but not spike time) information (Section 3.2) from these data will be harvested in model fitting. *In vivo*-like fluctuating-current test sets were probed within a wide range of SD, from 20 to 550 pA. For the preparation used here (adult rodent PFC), however, only the lower portion of this spectrum (SD of ~35–50 pA) produced voltage fluctuations in the recorded cells (σ*_V_* ≤ 6 mV, range ≤ 30 mV) that were most consistent with *in vivo* data [σ*_V_* ~ 1–5 mV, range ~ 10–20 mV, during awake activity or up-states as extracted from (Steriade et al., 2001; Timofeev et al., 2001; intracellular recordings); and (London et al., 2010; patch-recordings)]. To further verify these numbers, we also analyzed *in vivo* patch-clamp recordings from the (anesthetized) adult rodent PFC (kindly provided by Dr. Thomas Hahn, Central Institute of Mental Health and BCCN Heidelberg-Mannheim). The voltage SD during up-states ranged from ~2 to 6 mV (<σ*_V_*> ~ 3.5 mV; voltage range ~ 10–20 mV) in these data, in agreement with the values we have extracted from the literature. In contrast, fluctuating-current injections into adult PFC cells recorded *in vitro* with σ = 100 pA already resulted in voltage fluctuations (σ*_V_* ~ 11.9 mV, range ~ 45–50 mV) that clearly exceeded the range observed *in vivo*. Thus, fluctuating test stimuli with σ = 35–50 pA were deemed to be the ones most relevant to the *in vivo* setting, and hence most of the subsequent discussion will focus on this range.

### 3.1 Performance of the simpAdEx on electrophysiologically recorded pyramidal cells and interneurons

**Figure 4:**
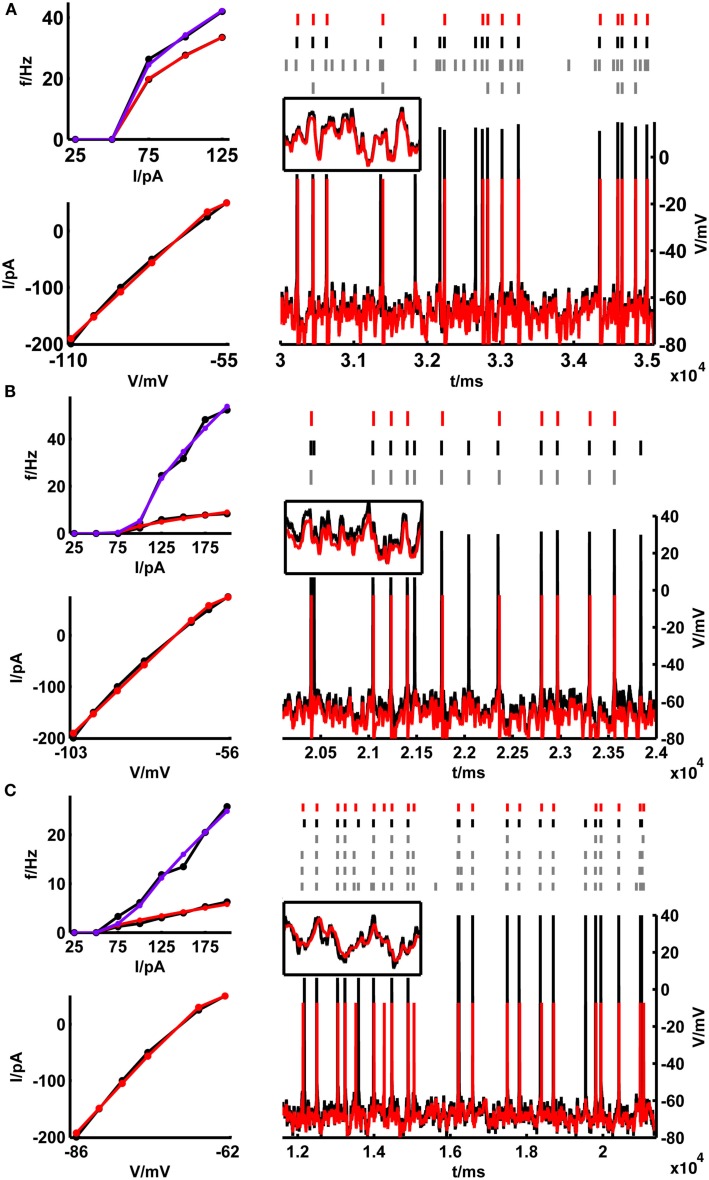
**Training set fits and example test set performance for (A) a bitufted interneuron (<Γ_mod_/Γ_cell_> = 1.2), (B) a layer-3 pyramidal cell (<Γ_mod_/Γ_cell_> = 0.78) and (C) a layer-5 pyramidal neuron (<Γ_mod_/Γ_cell_> = 0.9) from the rodent prefrontal cortex**. The training set consisting of the onset and steady-state *f*–*I* curves as well as the sub-rheobase *I*–*V* curve (left panel), and the test set (right panel) consisting of a voltage trace upon a fluctuating input current are given in black [**(A)** rheobase ≈ 50 pA, σ = 35 pA, μ = 15 pA; **(B)** rheobase ≈ 80 pA, σ = 50 pA, μ = 50 pA; **(C)** rheobase ≈ 50 pA, σ = 100 pA, μ = 25 pA]. The corresponding fits by the simpAdEx are given in red (steady-state *f-I* curve) and blue (onset *f*–*I* curve). The spike trains (top, right panel) illustrate the variability in the recorded cell responses to identical repetitions of the same fluctuating-current input (black and gray), together with the spiking responses of the model (red). Insets show zoom-ins on the subthreshold regime (t-interval: 500 ms, V-interval: 30 mV).

**Figure 5:**
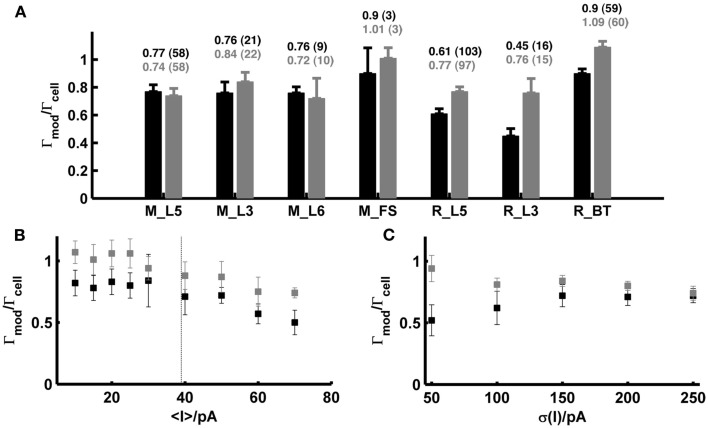
**Summary statistics for model performance on test sets from prefrontal cortical cells recorded *in vitro***. **(A)** Performance measure averaged across all test sets (σ ∈ {20,35,50} pA; in addition σ ∈ {100,150,200,250} pA for rat layer-5 cells) for different species, cell types and layers calculated by the normalized coincidence rate <Γ_mod_/Γ_cell_> for a window Δ = 20 ms without (black) and with (gray) a scaling factor that compensates for potential firing rate differences. The number of test sets investigated is given in parentheses. **(B)** Performance measure as a function of the mean input; points are averages across the two SD σ ∈ {35,50} pA [taken from the rat data shown in **(A)**] without (black) and with (gray) scaling factor. Dashed vertical: average rheobase of the cells shown. **(C)** Averaged prediction performance for layer-5 pyramidal cells from rats (*N* = 14 for σ = 50 pA; *N* = 6 for σ > 50 pA) as a function of the SD (mean current μ ≤ 25 pA) without (black) and with (gray) optimal scaling factor. All performances are given relative to the intrinsic reliabilities Γ_cell_ of the cells. Error bars = SEM. M_L5, mouse layer-5 pyramidal cell; M_L3, mouse layer-3 pyramidal cell; M_L6, mouse layer-6 pyramidal cell; M_FS, mouse fast-spiking interneuron; R_L5, rat layer-5 pyramidal cell; R_L3, rat layer-3 pyramidal cell; R_BT, rat bitufted interneuron.

Pyramidal cell (*N* ~ 90) and interneuronal (*N* ~ 10) recordings *in vitro* were obtained from layers 3, 5, and 6 of the adult rat or mice medial prefrontal cortex (see Materials and Methods). Figure 4 shows a few examples of training set fits and corresponding test set performances of the model on data from one bitufted interneuron (Figure 4A) and from pyramidal neurons in layers 3 (Figure 4B) and 5 (Figure 4C). The empirical *f*–*I* curves usually cover the whole range of spike rates up to the point of depolarization block (i.e., where spike-generating Na^+^ channels cannot recover from inactivation anymore) and were fitted very well in each case. Comparisons of the model and real cell test set voltage traces upon fluctuating currents also revealed an often remarkably high rate of precise spike coincidences (as in the examples shown), considering that these fluctuating test sets are very different from the data that had been used for adjusting the model. Even though the model spike reset points are often below the experimentally measured ones (see also Clopath et al., 2007; Badel et al., 2008b), the model V-traces are almost always able to make up with the original data before the next spike is reached. A missed or additional spike only leads to very transient deviations and has no longer-term effects. Thus, in general, spike times within the empirically recorded traces are predicted quite accurately. Although not a major objective of the present study, for the pyramidal cells also the correlations between model and measured subthreshold voltage traces (with spikes cut out in ±10 ms windows) were quite high, with a mean of 0.76 (*n* = 30 from 3 cells) when normalized to the intrinsic reliability of the empirical cells (i.e., the membrane potential correlations between different repetitions of the same trace). For fast-spiking interneurons these fits were substantially worse (normalized <*r*> = 0.36, *n* = 31 from 3 cells) for reasons discussed below (Section 4.3).

Figure 5A (black bars) summarizes all spike prediction results for different layers and cell types in rats and mice, across mean-input currents both above and below rheobase, and across a range of different SD (σ ∈ {20,35,50} pA; for rat L5 cells data contain in addition test sets with σ ∈ {100,150,200,250} pA). The performance measures were normalized by the intrinsic reliabilities of the cells as in previous studies (Jolivet et al., 2006, 2008; Clopath et al., 2007; Badel et al., 2008a,b; Naud et al., 2008; Gerstner and Naud, 2009), since in general the agreement between different spike train repetitions upon the same input from a real cell sets an upper bound on the prediction performance we may expect from a model. Only cells with intrinsic reliability ≥0.2 were considered in the present analysis (cf. Jolivet et al., 2008). When averaging across all the group means in Figure 5A, the coincidence rate Γ for a coincidence window Δ = 20 ms was about 0.74, and 0.64 for Δ = 10 ms [VP(*q* = 4/<ISI>) = 0.82; VP(*q* = 0.125 ms^−1^) = 0.67].

Figure 5B shows how the model performance depends on the mean input μ averaged over two different SD σ ∈ {35,50} pA: It seems that prediction performance is slightly better for lower μ close to or less than the rheobase. Thus, one may conclude that above the rheobase further mechanisms play a role that are not captured by the model that well. In particular, fluctuating currents with a large mean μ might contain more current events that drive the real cell close to or beyond the depolarization block. Hence, one explanation of the decreasing performance with higher μ might be that the model lacks an explicit Na^+^-channel inactivation mechanism. *In vivo*, however, spiking rates of prefrontal neurons are usually quite low even upon stimulus presentation (<10–20 Hz; Margrie et al., 2002; Lee et al., 2006; Lapish et al., 2008; Durstewitz et al., 2010), such that firing regimes beyond the rheobase and close to depolarization block may not be very physiological anyway (in fact, a common idea is that cortical neurons *in vivo* reside in a balanced regime right below the spiking threshold; van Vreeswijk and Sompolinsky, 1996; Destexhe et al., 2003; Renart et al., 2006). Figure 5C in addition shows that the prediction performance was also consistently high in layer-5 pyramidal cells across a wider range of variances for mean currents below the rheobase (μ < 25 pA).

Table 1 summarizes the parameter estimates for the simpAdEx for pyramidal cells in layer 3 and 5, and for fast-spiking interneurons.

**Table 1:** **Statistics of parameter estimates of the simpAdEx for different PFC layer 3 (L3) and 5 (L5) pyramidal cells (PC) and fast-spiking (FS) interneurons**.

Parameter	L3 PC	L5 PC	FS
*C* (pF)	123.71 (43.99)	213.94 (94.47)	54.72
*g_L_* (nS)	7.16 (2.41)	5.58 (1.71)	5.08
*E_L_* (mV)	−71.48 (6.71)	−71.42 (4.61)	−67.30
Δ*_T_* (mV)	4.51 (0.94)	2.80 (0.94)	2.93
τ*_w_* (ms)	120.98 (56.37)	218.07 (125.69)	22.23
*b* (pA)	19.82 (14.51)	19.65 (18.79)	2.04
*V_r_* (mV)	−84.23 (14.07)	−64.35 (5.15)	−100.03
*V_T_* (mV)	−55.38 (10.90)	−61.00 (10.90)	−53.97

*Values given as means (SD)*.

### 3.2 Optimizing spike-time prediction by compensating firing rate variations

**Figure 6:**
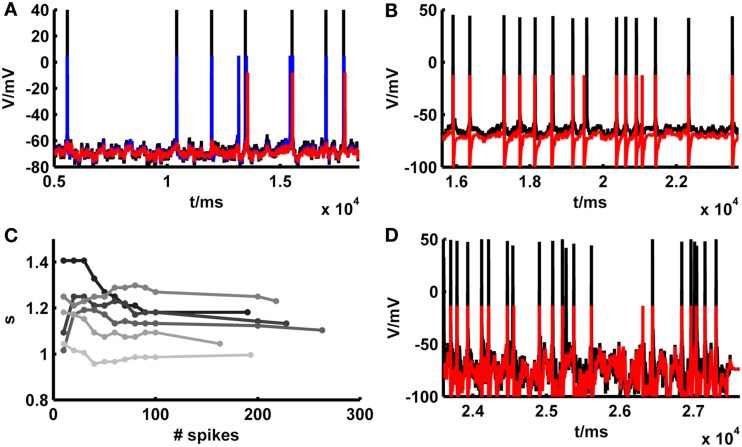
**Illustration of spike prediction performance under various conditions with and without scaling factor**. **(A)** Example where test set performance of the simpAdEx for a layer-5 pyramidal neuron (red; <Γ_mod_/Γ_cell_> = −0.02) is significantly improved by including a scaling factor (blue; <Γ_mod_/Γ_cell_> = 0.79). Original traces are given in black (σ = 50 pA, μ = 25 pA). **(B)** Performance of the simpAdEx on a test set with a scaling factor only adjusted on the first half of the fluctuating trace (not shown) for a fast-spiking interneuron from rodent prefrontal cortex (σ = 50 pA, μ = 50 pA). **(C)** The optimal scaling factor *s* as a function of the number of spikes used to determine *s*. Six different test sets are shown in different shades of gray for a layer-5 pyramidal neuron. **(D)** Test set performance of the simpAdEx for a layer-5 pyramidal neuron (without scaling factor) under high input variation modeled as an Ornstein–Uhlenbeck process (σ = 400 pA, μ = 25 pA).

Although, as shown in the previous section, in many cases the simpAdEx model adjusted purely on the basis of constant-current-step protocols performed quite well on the independent fluctuating test sets, in other cases the model appeared to match the empirical spike trains less well (Figure 6A). We noticed, however, that these cases most often were not due to an inability of the model to capture the empirical spike times *per se*. That is, whenever the model and the real cell both elicited a spike around a particular time, these were often precisely aligned (as Figure 6A illustrates). Rather, the total number of spikes elicited by the model and real cells deviated in these situations (the source of these deviations will be investigated further below), and as a consequence of this some of the spikes produced in one preparation (model or real cell) had no counterpart in the other. Therefore, to examine to which levels model performance could be pushed based on firing rate information alone, a constant scaling factor *s* for the input *I*(*t*) [see equation (20)] was introduced. This multiplicative factor was determined solely by matching the total number of spikes within the “recording periods” of our simple model and of the physiological cells, that is without any other adjustments that would aim to capture the precise spike times (other parameters of the model could be scaled instead, e.g., E*_L_*, which comes down to adjusting an additive constant). As exemplified in Figure 6A, compensating the mismatch in model and empirical test trace firing rates by setting the scaling factor suffices to bring the spike trains to almost perfect agreement. The gray bars and markers in Figure 5 summarize the results across all data sets also studied in the previous section, demonstrating that in general the introduction of a scaling factor based solely on firing rate information improves spike-time predictions. With a scaling factor in place, Figure 5 also contains average performance measures slightly above one [formally the coincidence rate defined by equation (45) can indeed be greater than 1; see Naud et al., 2011]. This indicates that adjusting the scaling factor on the test traces may result in model – real cell agreements which are higher than the agreement between different repetitions from the same real neuron, as explored further below. One may argue, of course, that our “test sets” in this case are not true test sets anymore, as still information from these traces (spike count) was used to adjust one of the model parameters. For achieving a comparable level of spike-time prediction, however, it is also sufficient to adjust the scaling factor just on an initial segment of the fluctuating trace, and then use the remaining trace as a truly independent test set (example in Figure 6B). As Figure 6C shows, the estimate for the scaling factor quickly converges after a few dozen spikes. Consequently, if any information from fluctuating traces may be harvested at all, prediction performance can still be driven to very high levels on test set bits not used at all for adapting model parameters (the conditions required in the INCF contest proposed by Gerstner and Naud, 2009).

**Figure 7:**
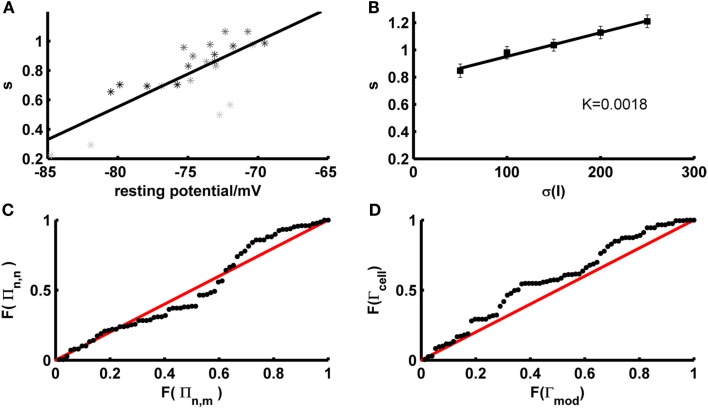
**Determinants and implications of the scaling factor in model prediction performance**. **(A)** The optimal scaling factor as a function of the experimentally recorded resting potential for 4 example test sets (gray to black shaded asterisks). Asterisks with she same shade of gray belong to identical repetitions of the same stimulus. **(B)** The optimal scaling factor as a function of the SD σ averaged across five layer-5 pyramidal cells (mean current μ = 25 pA). K = slope of the linear fit. **(C)** P–P plot of the distributions *F* of the firing rate agreement measure between model and neuron Π_*n*,*m*_ (without scaling factor) and the firing rate agreement measure between different trials of the neuron Π_*n*,*n*_ for a subset of the data (σ ∈ {35,50}, μ ≤ rheobase). The line *F*(Π_*n*,*n*_) = *F*(Π_*n*,*m*_) is given in red. **(D)** P–P plot of the distributions *F* of the model performance Γ_mod_ (with scaling factor) and the cell reliability Γ_cell_ for a subset of the data (σ ∈ {35,50}, μ ≤  rheobase). The line *F*(Γ_mod_) = *F*(Γ_cell_) is given in red.

One potential factor that may contribute to the firing rate deviations compensated by the scaling factor are the fluctuations that naturally occur across different experimental repetitions of the test trace *in vitro*. In fact, we observed that the firing rates during different identical fluctuating test repetitions within the same experimentally recorded cell systematically depend on the resting potential right before the test set application [both overall positive (<r> ~ 0.6, *p* < 5 · 10^−32^) and negative (<r> ~ −0.56, *p* < 10^−18^) correlations were observed; across all sets: <r^2^> ~ 0.43, *p* < 0.002 according to a permutation bootstrap test]. This suggests that the scaling factor may partly compensate for experimental noise, that is fluctuations in precise resting conditions (potentially associated with variations in ionic milieu that occur across time) which cause corresponding fluctuations in cell excitability and firing rates. Indeed, not surprisingly, just like the empirical firing rates, the magnitude of the scaling factor needed was significantly correlated with the experimentally recorded resting potential just before test set application (Figure 7A; <|*r*|> ~ 0.59, *p* < 10^−20^; <r^2^> ~ 0.43, *p* < 0.002 according to permutation bootstraps). In our data sets this source of experimental noise may have been particularly problematic because, as already noted above, we have mostly employed lower SD (≤50 pA) for the fluctuating-current inputs than used in most previous studies (≥150 pA; e.g., Clopath et al., 2007; Badel et al., 2008b).

Hence, the impact of variations in resting conditions relative to the voltage fluctuations caused by the stimulus may have been higher than in many previous studies. This is also the reason why we had to allow for somewhat broader time windows (10–20 ms) for detecting spike coincidences than in many previous studies (with time windows more in the range of 5–10 ms). To investigate this issue of comparability with previous studies further, four layer-5 PFC pyramidal cells were recorded using *I*_inj_ SD ranging from 250 to 550 pA (cf. Badel et al., 2008a,b) and implementing precisely the same type of random process (Ornstein–Uhlenbeck process) as most frequently employed previously (cf. Rauch et al., 2003; Clopath et al., 2007; Badel et al., 2008a). In these cases, reasonable prediction performance (without including or adjusting a scaling factor) was indeed achieved on the test sets for a coincidence window of only 5 ms [Figure 6D; <Γ> = 0.6, max(Γ) = 0.83].

Another potential source of the firing rate deviations may be that our training approach does not include any samples explicitly representing input variation, unlike fitting approaches directly based on fluctuating traces. It is known that the spike rate of neurons does not only depend on the mean input but as well on its variance (Mainen and Sejnowski, 1995), with the result that the shape and slope of *f*–*I* curves can change considerably with the input (Chance et al., 2002; Rauch et al., 2003). Information about variance-dependency of neural spiking is not explicitly represented in our training set data, although it may come in implicitly through the fact that both steady-state and *transient* *f*–*I* information is used. To investigate the contribution of the input variance in the test sets with mean currents less than the rheobase, the required scaling factor was plotted as a function of the input variance across empirical data sets (*N* = 5 cells) for which a larger range of SD had been probed. As shown in Figure 7B, the scaling factor is approximately linear in the SD σ, with a relatively shallow slope (~2 · 10^−3^/pA). Hence, at least within the more physiological regime of below-rheobase mean inputs, its dependence on the input variance seems not too high, suggesting that at least part of the mechanisms accounting for the variance-dependence of spike rates may have been implicitly captured by our training sets and intrinsic properties of the model. Also note that this slope factor implies that for a range of SD that may be considered physiological based on the *in vivo* analysis preceding Section 3.1 (σ*_I_* ~ 25–50 pA), the scaling factor may vary by no more than ~5% across this *in vivo* range (in contrast to the variation caused by different resting conditions, Figure 7A). The conclusion that input variance has a comparatively mild effect on model performance is further reinforced by the observation that spike-time prediction performance itself does not strongly depend on input variance [Figure 5C; if anything, it tends to slightly increase with higher variance and no input scaling (black markers)].

The analyses above demonstrated that spike-time prediction could be further improved by just compensating for total spike count differences between model and target traces through a scaling factor, and have identified potential experimental sources for the firing rate deviations. For an application of our model to *in vivo* situations, a crucial question therefore is how severe or limiting these firing rate deviations between model and empirical traces actually are in relation to the physiological variation observed under *in vivo*-like stimulation conditions (current σ ≤ 50 pA, μ < rheobase; without scaling factor, the overall agreement under these conditions for cells with intrinsic reliability Γ_cell_ ≥ 0.2 was <Γ> ~ 0.73 for a coincidence window of 20 ms). We therefore determined the agreement in firing rates (*f*) by

(47) $$\prod_{n,m}=1-\frac{\text{|}f_{n}-f_{m}|}{\left(f_{n}+f_{m}\right)}$$

with “*n*” denoting neural and “*m*” model firing rates. This was done for various repetitions of the same current input to the same cell as recorded physiologically (Π_*n*,*n*_), and between the model traces (without additional scaling) and the physiological recordings (Π_*n*,*m*_). As already noted in Section 3.1, model predictions should be accepted as reasonably good if they lie within the range of empirical variation, that is compared to the agreement between different spike train repetitions from experimentally recorded cells under exactly the same input conditions. To quantify this relation, the distribution of model-real cell firing rate agreements Π_*n*,*m*_ was compared to the distribution of real cell-real cell agreements Π_*n*,*n*_ using a percentile–percentile (P–P) plot (Figure 7C). The P–P-graph places data points at coordinates corresponding to the percentiles of the model-real cell (abscissa in Figure 7C) and the real cell-real cell (ordinate in Figure 7C) distributions *F* into which these points fall. If the two distributions were exactly the same, the P–P plot would follow the line *F*(Π_*n*,*n*_) = *F*(Π_*n*,*m*_), while it would range above it if model predictions were better than empirical reliability and below it if they were worse. The graph contains all test sets with mean currents μ below the rheobase and SD σ ∈ {35,50} pA. As Figure 7C (black dots) demonstrates, the model-real cell distribution is well en par with the real cell-real cell distribution, and – if anything – tends to actually range above it for the higher percentiles. This indicates that the model-empirical firing rate agreements are at least as good as the reliability among different identical-input-repetitions from the same cell, i.e., almost optimal if set in relation to the empirical variation. That the model firing rate agreements tend to be actually slightly better than the experimental reliability might be explained by the fact that the models are fit to *f*–*I* training sets close in time to the corresponding test sets (relative to the temporal spacing between different test sets). Therefore, they may already account for some of the factors causing variation across different experimental repetitions (across time; see above), and hence perform even slightly better than expected from the empirical variation.

We may also conjecture from these observations that explicitly adjusting an input scaling factor to precisely match empirical and model trace spike counts could actually result in over-fitting, that is adjusting the model toward part of the empirical noise rather than capturing the true expectancy of the empirical distribution. This is indeed confirmed by the P–P-plot shown in Figure 7D, which illustrates that the distribution *F* of spike-time agreements Γ_mod_ between the model *including* a scaling factor and the real cells is actually shifted to consistently higher values compared to the distribution between different repetitions of the same fluctuating stimulus within the same cell (Γ_cell_). That is, for any level of agreement between two spike trains there are more model-real cell coincidence rates ranging above that level than real cell-real cell coincidence rates, indicating that the model with scaling factor performs better than would be expected from the experimental distribution.

In conclusion, the simpAdEx model adapted solely based on conventional step protocols performs well in *in vivo*-like test situations within the bounds of physiological reliability. Inclusion of the scaling factor demonstrates to which levels of precise spike-time performance the simple model could theoretically be stretched relying only on firing rate information for training (plus sub-rheobase *I*–*V* curve). However, given that it may also lead to over-fitting, for practical application of the model to *in vivo* network situations inclusion of this additional parameter is not advisable.

### 3.3 Comparison to the adaptive LIF model and the full AdEx

**Figure 8:**
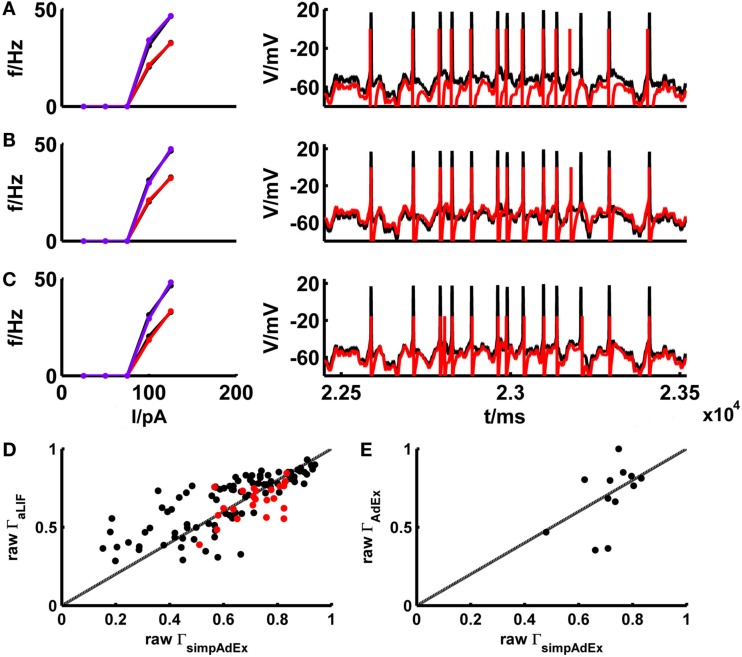
**Comparison of the simplified AdEx (simpAdEx) with the adaptive LIF neuron (aLIF) and the full AdEx**. The onset and steady-state *f*–*I* curves and the test set consisting of a voltage trace upon a fluctuating input current for a fast-spiking interneuron from the rodent prefrontal cortex are given in black. The corresponding model fits [**(A)** aLIF, **(B)** simpAdEx, and **(C)** AdEx] are given in red (steady-state *f-I* curve) and blue (onset *f*–*I* curve). **(D)** The raw coincidence rate Γ for a window Δ = 20 ms for 170 evaluated test sets from 39 cells (layer-3 and -5 pyramidal cells, fast spiking and bitufted interneurons) for aLIF and simpAdEx. Black dots = σ ∈ {35,50}, red dots = σ ∈ {100, 150,200,250}. **(E)** Raw Γ for a window Δ = 20 ms for 12 evaluated test sets of 5 cells (see Table 2) for AdEx and simpAdEx. A scaling factor for eliminating firing rate differences was included in all model comparisons (see text).

The basic LIF model enjoys great popularity when it comes to larger-scale network simulations due to its mathematical simplicity and computational efficiency. In Materials and Methods we derived (exact) closed-form expressions for the onset and steady-state firing rates of the adaptive LIF model, that is the basic LIF model enhanced by a second linear differential equation implementing a spike-rate adaptation process (see Materials and Methods: 2.2.2 and 2.2.3). The parameters of the model are tuned by the same fitting algorithm on training sets consisting of *f*–*I* and *I*–*V* curves as used for the simpAdEx (see Materials and Methods: 2.3). To minimize the effect of experimental noise and enhance comparability between models, potential mismatches in spike counts between model and target traces were largely eliminated through input scaling as introduced in the previous section. This way the adaptive LIF was adjusted for a subset of 39 different real cells, and its performance on the corresponding fluctuating-current test sets was compared with the one from the simpAdEx. Figure 8 gives the training and test result for an example of a physiological cell. The aLIF like the simpAdEx can be seen to reproduce spike times with high accuracy, although the aLIF clearly does not capture the subthreshold dynamics as well. Figure 8D shows the (non-normalized) spike coincidence rates (gamma factors) for the aLIF versus simpAdEx models for different test sets. Despite the further simplification in the aLIF model, its spike prediction performance, once firing rate differences were eliminated, is comparable to the simpAdEx for small SD (black dots), while for higher input variance (red dots) the coincidence rates are below those from the simpAdEx.

The development of the approximation to the full AdEx was motivated by the closed-form expressions that could be derived for the training set, and the much faster fitting procedure implied by this (see Materials and Methods). The considerable speed-up in fitting times is verified in Table 2 which summarizes for five different physiological neurons the computer time needed to fit the three training set curves for the full AdEx with (*a* ≠ 0) and without (*a* = 0) subthreshold adaptation, and for the simpAdEx. The parameters of the full AdEx are tuned by the same fitting algorithm used for the simpAdEx (see Materials and Methods: 2.3), only that numerical integration of the differential equations was required for the full AdEx due to the lack of closed-form *f*–*I* expressions. These data evidence speed-ups of 1–2 orders of magnitude, as noted in previous sections. In terms of spike-time prediction, the comparison of (non-normalized) coincidence rates for 12 test sets from 5 cells is given in Figure 8E (again with input scaling present to eliminate firing rate deviations as a contributing factor), and a specific example of training and test set fit is shown in Figure 8C. On average, the performance does not seem to increase by using the full instead of the simplified AdEx.

**Table 2:** **Comparison of computation time requirements for the full and simplified AdEx**.

Cell type, species	t/min AdEx (*a* = 0)	t/min AdEx (*a* ≠ 0)	t/min simpAdEx
L5, rat	332	900	12
L3, mouse	248	850	7
FS, mouse	215	370	3
L5, mouse	305	860	6
L5, mouse	400	778	13

## 4 Discussion

For the purpose of large-scale neuronal network simulations (Traub et al., 1988, 2005; Markram et al., 2004; Markram, 2006; Wang et al., 2006; Izhikevich and Edelman, 2008; Lansner, 2009), single neuron models which do not compromise physiological realism too much and capture some of the tremendous cellular heterogeneity observed in real cortical tissue are of increasing interest. Here we introduced a novel approach for fitting a simple 2-ODE neuron model to experimental data with good prediction performance on distinct test sets not employed for fitting (Section 3.1). Our approach had several important objectives: (1) We wanted the fitting procedure to be fast (cf. Table 2) and completely automatized (see Materials and Methods), so that large pools of neural recordings could easily be translated into single-cell models; (2) we only wanted to rely on simple standard electrophysiological protocols for this process, i.e., *f*–*I* and *I*–*V* curves, which are widely available and routinely obtained; (3) the training set for model fitting should assess a wide range of firing rates (as rate changes are still the most prominent responses correlated with behavior observed *in vivo*); (4) the models should exhibit satisfactory prediction performance on physiological recording sets distinct from the ones used for training, in this case spike responses upon fluctuating “*in vivo*-like” current injections.

Toward these goals, we developed an approximation to the AdEx model from which closed-form expressions for initial and steady-state *f*–*I* curves could be derived. For the AdEx model, originally introduced by Brette and Gerstner (2005), it has been shown previously that it can reproduce a variety of spiking patterns observed in diverse cell types (Naud et al., 2008), thus forming a good starting point for our own analysis. Our approximation to the AdEx was based on separation of time scales and phase plane considerations. Since we do not have to solve for the full model trajectory up to a steady-state by numerical integration but can directly calculate the *f*–*I* curves from closed-form expressions, the fitting procedure is sped up by about two orders of magnitude (cf. Table 2). This allows to construct large sets of model neurons from empirical data sets conveniently and quickly. Based on our analytical approximation, one can also easily find closed-form expressions for, e.g., the latency to the first spike or the number of spikes in response to a step current.

There are, of course, also other approaches to fast model fitting. Progress in global optimization techniques and computer hardware substantially decreased the temporal requirements for fitting models to experimental data (Brette et al., 2007). Evolutionary techniques (Bäck and Schwefel, 1993) like genetic algorithms, differential evolution and related methods, or particle swarm algorithms (Eberhart and Shi, 1998), enable fast optimization of multi-dimensional systems by efficient parallelization, in particular through the use of graphics processing units (GPU; Owens et al., 2007; Rossant et al., 2010). Other algorithmic solutions like implementations based on vectorization, that is replacing multiple repeated operations by single operations on vectors, can make optimization processes more efficient (Brette and Goodman, 2011). For instance, in one such recent approach (Rossant et al., 2011) 50–80-fold speed improvements were found combining vectorization techniques and parallelization on 240 GPU cores when compared to run times on a single GPU. Most of these algorithmic and hardware approaches may also be harvested for our model, however, to further speed-up the process if many different cell types are to be fitted at once. The advantage of our approach lies primarily in alleviating the need for explicitly simulating the system of differential equations. The optimization function equation (44) may then still be subjected to evolutionary, swarm, or vectorization techniques, or different model cells from a larger data set may be optimized in parallel on different CPUs/GPUs.

Our AdEx approximation can still reproduce most of the spiking patterns of the original AdEx, and thus many of the patterns observed in real neurons. As an even simpler alternative, we also considered an adaptive LIF model for which we derived exact closed-form expressions for the onset and steady-state firing rates. Both models could be easily fit to near perfection to the initial and steady-state *f*–*I* curves of recordings from real pyramidal and interneurons from various layers of the rodent prefrontal cortex. The simpAdEx model on top captured sub-rheobase *I*–*V* curves very well.

### 4.1 Nature of the training set: Limitations and extensions

The major advantages of the training set used for our modeling approach is its simplicity and easy availability, and, of course, the fact that it allows for the fast fitting procedure based on closed-form expressions. In contrast, for the purpose of model fitting and evaluation, most previous studies have employed fluctuating currents designed to reflect the synaptic bombardment by populations of excitatory and inhibitory presynaptic cells (Jolivet et al., 2006, 2008; Clopath et al., 2007; Badel et al., 2008a,b; Naud et al., 2008; Gerstner and Naud, 2009). Unlike fitting procedures working on such fluctuating current/voltage traces, our training set probes none of the higher-order statistical properties (at least not explicitly, see above) that may characterize *in vivo* activity. In this sense it is more removed from the *in vivo* scenario to which the model is ultimately to be applied to, than training sets used in previous approaches. However, it may be important to note that the construction of *in vivo*-like fluctuating training or test sets rests on many assumptions about synaptic dynamics, amplitudes and time constants, input rates, correlations among inputs, frequency content, and so on. Prediction quality on real *in vivo* situations is likely to depend on how well these assumptions are met, and like it is generally true in statistics (e.g., Hastie et al., 2009), one may sometimes be better off using simpler methods making less assumptions. Furthermore, due to the time constraints imposed by *in vitro* methods (limited life time and intracellular integrity/composition of the cells), only a limited parameter range can be probed by any training protocol for a given cell, such that results could be biased toward the specific parameter regime explored. For instance, while our training protocol contains a large range of mean-input currents and thus spike output rates a neuron may traverse *in vivo*, up to the point of depolarization block, this was not always the case in all of the previous approaches based on fluctuating inputs. For these reasons, it may not be *per se* clear that any fitting procedure based on fluctuating input currents would also automatically transfer better to an *in vivo* situation. This is not to say that a training protocol working with step-like inputs is actually to be preferred, but just to caution that generalization performance will likely depend on the details of the implementation of the fluctuating inputs and the parameter range probed. Ideally, of course, the better an approximation one can get to the *in vivo* situation, and the more data sets for covering the “*in vivo* space” one has, the better this would be for training a model.

One other issue that deserves discussion in this context is the similarity between training and test sets, and the fitting criterion used. If both training and test data consist of noisy voltage traces which in addition may be generated from similar underlying distributions, then while test set prediction performance should be better than if dissimilar training data were used, generalization to completely different scenarios may be worse (see also discussion on within- vs. out-of-sample predictions in, e.g., Hastie et al., 2009). This may be exaggerated if the fitting criterion explicitly includes the specific quantities to be predicted, i.e., the precise spike timing in the present case (for instance, some fitting criteria that have been in use directly included the spike-time agreement measure Γ as a term). Our approach uses training and test sets which are very distinct: On the training side, we used subthreshold *I*–*V* and onset and steady-state *f*–*I* curves based on step-like inputs, while the test sets consisted of fluctuating *in vivo*-like current inputs of different means and SD. The fitting criterion based on the *I*–*V* and *f*–*I* curves also did not involve any spike-time information (the target of prediction). In this sense we feel that our test sets impose a quite strong generalization test on the model. However, this discussion may boil down to the same issues already brought up above: If much knowledge about the ultimate application domain is available and can be integrated into the training sets, then it should certainly be used, and fitting criteria that actually emphasize those aspects on which high prediction performance is sought, may be preferred. In this sense this discussion is less a critique of the various fitting approaches (including ours), but more to propose this whole subject as important for more detailed future research.

As noted above, our training set composed of steady-state and transient *f*–*I* curves does not directly assess higher-order statistical properties of the input. With regards to the input variance, one simple potential extension is to augment the set of constant hyperpolarizing and depolarizing current steps with step-like positive or negative excursions from any given level of injected current. Such an approach may allow to assess in more detail how a cell responds to transient mild or larger deflections from any mean-input level (i.e., to variations of different size). At the same time it still largely retains the simplicity of experimental protocols consisting only of current-clamp steps and thus also retains the possibility to fully specify the model by using only steady-state and transient *f*–*I* curves, an important feature of the fitting procedure introduced here. Within the limits of the resolution required to obtain estimates of the instantaneous firing rate (at least one interspike interval), the frequency of these step-like changes may also be varied to assess some of the frequency-dependent aspects of the neural response while still enabling model specification via *f*–*I* curves.

### 4.2 Prediction performance, input regimes, and firing rate variations

To assess the predictive power of our modeling approach, a large set of data from purpose-generated electrophysiological recordings from different cell types and cortical layers (L3, L5, and L6) was analyzed. Prediction performance was formally evaluated using two measures (coincidence rate and Victor–Purpura measure) which quantify the accuracy in predicting spike times within the test sets (our results, however, did not strongly depend on the employed performance measure or its parameter settings). Since previous fitting procedures with reduced models most often used fluctuating input stimuli both for the training and the testing (see Section 4.1), and furthermore often optimized the coincidence rate directly (e.g., Clopath et al., 2007), it seems natural that they should have an edge compared to our approach in predicting precise spike times within the test traces. However, prediction performance in our model (Section 3.1) seems quite en par with levels reported previously for different sets of probed input conditions (compare, for instance, to the two-compartment model based on the AdEx presented in Clopath et al., 2007, or the refractory exponential IF model in Badel et al., 2008a,b), and could be improved further by introducing a constant scaling factor for the current input into the voltage equation of the simpAdEx model (Section 3.2), adjusted to level the total spike counts in the model and reference traces. Allowing further adjustment through this single scalar scaling factor led to often almost perfect agreement of spike trains, although it was tuned solely to match the total number of spikes in the model and target traces, that is without using detailed spike time or voltage information. It was also sufficient to use only a portion of the fluctuating test traces for adapting this parameter (cf. Figures 6B,C), leaving the remainder of the fluctuating voltage trace as a truly independent test set. However, we found that the scaling factor may actually harm generalization performance, as it leads to over-fitting (Figure 7D), and for most practical purposes (*in vivo* scenarios) should therefore not be included in the model, as further discussed below.

Nevertheless the results surrounding the scaling factor highlight a number of interesting and important points about model fitting, dynamics, and empirical noise. First, it is interesting to note that in any step of the model fitting approach, including adjustment of the scaling factor, only firing rate (plus sub-rheobase *I*–*V*) information has been used, yet the model is able to predict quite accurately precise spike times for a whole range of different input scenarios. This suggests that, for the situations tested, the underlying spike-generating dynamics of complex real neurons was properly captured by our simple 2-ODE model. More importantly, it indicates that – once basic dynamical properties are captured – all the information about spike times is already contained in the firing rates (and possibly vice versa), potentially shedding some new light on the relation between firing rate and precise spike-time codes (Shadlen and Newsome, 1994; Gütig and Sompolinsky, 2006; London et al., 2010). Second, these analyses revealed factors from where model – real cell differences may come from. One idea was that firing rate deviations could potentially arise from the fact that neural spiking rates do not just depend on the mean but also on the variance in the input current (Mainen and Sejnowski, 1995; Chance et al., 2002; Rauch et al., 2003). Our training set does not explicitly contain voltage traces of different variance. However, it was found that at least below the spiking threshold, presumably the regime in which neurons operate *in vivo* (Destexhe et al., 2003; Renart et al., 2006), the scaling factor did not change too much (with a slope of ~2 × 10^−3^/pA) across a large range of input variances (cf. Figure 7B), nor did spike prediction performance itself (Figure 5C). Potentially this is because at least part of the relevant information is still available to the fitting process through the transient *f*–*I* curves. Neocortical neurons *in vivo* have indeed been proposed to live in a subthreshold balanced regime where spiking is caused by occasional threshold crossings (van Vreeswijk and Sompolinsky, 1996; Destexhe et al., 2003; Renart et al., 2006), an idea consistent with the relatively low firing rates of neocortical neurons *in vivo* (Margrie et al., 2002; Lee et al., 2006; Lapish et al., 2008; Durstewitz et al., 2010).

Another idea was that model – real cell firing rate deviations may in part simply reflect empirical noise, that is firing rate fluctuations that naturally occur across different identical repetitions of the same stimulus conditions in the recorded neurons (Mainen and Sejnowski, 1995). Indeed, firing rates recorded for identical stimulus applications significantly co-varied with fluctuations in the cellular resting potential preceding the application, and so did the scaling factor (cf. Figure 7A). This problem may be exaggerated with larger temporal gaps between the training and test sets (as in the present study, ~5 min on average), as during longer time periods the precise physiological conditions during test set application may be more likely to have drifted away from those during training set application. It may also be more of a problem in slice preparations from adult animals as compared to the much more widely used juvenile preparations. Adult slices exhibit richer intrinsic activity and less stability than juvenile slices (e.g., Tseng and O’Donnell, 2005). In contrast, it should be less of a problem if the variations in the input are large (e.g., σ = 250–550 pA, as in Figure 6D). Thus, fluctuations in physiological background conditions and the signal/noise-ratio are factors that influence the model fitting process (see also below). These results may also have implications for neural processing *in vivo*: Either synaptic events have to be of sufficiently large amplitude to overcome background fluctuations and cause reliable spike timing (see London et al., 2010), or variations in background conditions need to be sufficiently common to the neurons supposed to communicate via precise spike times (a condition that in the model – real cell comparisons could be installed through the scaling factor).

The physiological noise also limits the degree to which spike times from one stimulus presentation can be predicted from another identical stimulus repetition in the same cell. As the model cannot be expected to perform better than the real cells, the statistical distributions of the empirical firing rate reliabilities (i.e., spike-rate agreements across different repetitions) and the model-real cell spike-rate agreements were first compared. For test sets with SD up to 50 pA (the *in vivo* range, see Section 3), this comparison revealed that the model’s firing rate accuracy was perfectly within the bounds of empirical variation (cf. Figure 7C). This means that inclusion of a scaling factor in the model on average does not truly improve the model – real cell fit beyond the degree of spike-rate agreement to be reasonably expected from the experimental variability. This in turn suggests that the scaling factor may in fact partly fit the empirical noise (i.e., lead to over-fitting of particular test traces), as was confirmed when the distributions of cell-cell and model-cell spike coincidence rates were compared: With the scaling factor in place, the model outperformed the real cells, that is led to spike coincidence levels that were consistently higher than those between different repetitions from the same cell (cf. Figure 7D). Thus, the scaling factor demonstrates the spike-time prediction power that can be achieved while exploiting only rate (plus sub-rheobase *I*–*V*) information as a source for model fitting. But the actual recommendation to be derived from this analysis is that none such additional parameter adjustment should be performed for application of the model to *in vivo*-like situations.

Finally, we would like to comment on the time windows used to evaluate spike coincidences (Γ) for most part in the present study. We have reported results for both 10 ms, which is within the range of 5–10 ms employed by most previous studies (Badel et al., 2008a,b; Jolivet et al., 2008), and 20 ms. The choice of the larger window of 20 ms was motivated by the fact that in most of our cells we had used lower SD for the fluctuating currents (≤50 pA) than most commonly used previously (≥150 pA; Jolivet et al., 2006, 2008; Clopath et al., 2007; Badel et al., 2008a,b; Naud et al., 2008; Gerstner and Naud, 2009). These lower SD, in turn, were adopted because they provoked voltage fluctuations most compatible with the range observed *in vivo* for our preparation (adult rodent prefrontal cortex; see Section 3). In ours as in previous studies (Mainen and Sejnowski, 1995; Chance et al., 2002; Rauch et al., 2003), cells become less reliable in their spiking patterns as the input variance decreases, which in turn makes the choice of larger time windows more reasonable (ultimately, it is of course the voltage variance most relevant here, but for a given set of cellular parameters this will be highly correlated with the current variance). When we used very high current variances for the input (250–550 pA, as, e.g., in Badel et al., 2008a,b) and the same type of random process for generating the fluctuations as used by others before (Ornstein–Uhlenbeck process; e.g., Rauch et al., 2003; Clopath et al., 2007; Badel et al., 2008a), spike coincidence levels between model and real cells approaching those reported previously (e.g., <Γ> = 0.6 as in Clopath et al., 2007) were obtained (without scaling factor) also for smaller time windows (5 ms). It may also be important to note that larger time windows do not necessarily imply higher coincidence rates: In Jolivet et al. (2008), for instance, the coincidence rate Γ was shown to be approximately constant for coincidence windows Δ ∈ [2 ms, 12 ms], and decayed for both smaller and larger time windows. Finally, it should be noted that different studies have employed different recording protocols, different parameter settings for generating current fluctuations, and different physiological preparations and cell types, which may limit comparability among studies (even when intrinsic cell reliability is used for normalization).

### 4.3 Subthreshold membrane dynamics, comparison between models and possible extensions

The main goal of our study was to develop a simplified neuron model which, when adjusted based on standard *in vitro* protocols, captures the spiking behavior (rate and timing) of real cells, as these are the quantities which represent the ultimate read-out of a neuron’s information processing accessible to other neurons, and are the quantities usually reported in *in vivo* studies. We observed, however, that on average the adapted simpAdEx model would also reproduce the subthreshold membrane voltage quite well (see insets in Figure 4). For prefrontal pyramidal cells, correlations with the membrane potential in the subthreshold regime were on average <r> ~ 0.58 (and increased to 0.65 with input scaling), just slightly below the range of cross-correlations observed between different repetitions of the same stimulus *in vitro*, <r> ~ 0.75. The main deviations from the voltage trajectory of real cells seemed to occur right after a spike where the model cells sometimes produced a considerable after-hyperpolarization while the real neurons often returned to a potential not far below the pre-spike level. This is to be expected, as the model cells have an artificial reset process and lack the ionic mechanisms (Na^+^-channel inactivation, diverse Ca^2+^- and K^+^-channels) that drive spike-after-polarizations *in vitro*. Note that this balance of ionic currents in the direct aftermath of a spike is not captured by the subthreshold *I*–*V* curves used for model fitting. This mismatch in after-spike voltage behavior has been described by others before for this class of simple models (e.g., Clopath et al., 2007; Badel et al., 2008b), and it may also be the reason why the subthreshold voltage correlations with interneurons [<r> ~ 0.27 (0.3 with input scaling), compared to a correlation of 0.75 among repetitions] are considerably worse than those for pyramidal cells (see above). Especially for fast-spiking interneurons the subthreshold voltage trajectory is dominated to a large degree by the post-spike polarizations which are not properly captured by the model. We emphasize that strongly reduced models like the present which are mainly adapted to capture the spiking behavior of real cells may not necessarily also exhibit good fits to the subthreshold membrane behavior (as actually evidenced by the aLIF example in Figure 8A). In some cases, they may even have to compromise voltage dynamics to achieve a good approximation to the spiking behavior. For spiking, the dynamics of the membrane voltage in the vicinity of (or approach toward) the threshold is most crucial. If the voltage dynamics is of primary interest, biophysically more detailed models may often be better suited, although we note that recently simple models have been advanced that also capture post-spike voltage behavior very well (Badel et al., 2008a,b; Rossant et al., 2010).

For several data sets, we have compared performance of the aLIF, the simpAdEx, and the full AdEx models (cf. Figures 8D,E). The aLIF is the simplest type of model that would allow reasonable fitting to our training data, as an adaptation term is essential for simultaneously matching the transient as well as the steady-state *f*–*I* curves. With a scaling factor allowed, the level of spike-time agreement (Γ) with the experimental cells was comparable for all three types of reduced models. However, experimentally recorded *I*–*V* curves often significantly deviated from linearity as the membrane potential approached the spiking threshold. Since the aLIF model is strictly linear in its current-voltage-relationship below threshold, it is not able to capture this experimentally observed departure from linearity. Likely for this reason, its agreement with the subthreshold membrane dynamics was often substantially worse than that of the simpAdEx model (compare Figures 8A,B). The fitting process for the aLIF model also often resulted in rather unphysiological parameter estimates (for instance, *V_r_* < −150 mV), probably to compensate for the mismatch in the subthreshold voltage dynamics when reproducing the spiking behavior. Thus the aLIF model (with scaling factor) does a reasonably good job in reproducing empirical firing rates and spike times, but the addition of an exponential term to the voltage equation seems to substantially improve the physiological validity of the model with regards to the membrane dynamics. Comparing the simpAdEx and the full AdEx model, the constraint that adaptation in the simpAdEx is solely driven by the reset since *a* = 0 may present a limitation for fitting physiological data in which subthreshold adaptation currents, like *I_ks_* (Hammond and Crépel, 1992), play a prominent role. However, in practice, it was often taken to be zero because it proved to be difficult or impossible to extract a reasonable value from electrophysiological recordings (Clopath et al., 2007). In general, it is important to keep in mind that all the models discussed here represent massive reductions of a much more complex physiological reality. Many if not most of the parameters in such models will therefore inevitably ultimately represent lumped contributions of different ionic sources or spatial factors. In this sense, in most cases there will be no 1:1 mapping on biophysical parameters of a real cell.

More generally, of course, as with every highly simplified neuron model, ours also ignores the spatial (dendritic) structure of real cells. One potential extension may be to add dendritic transfer/filter functions to the model which allow to fit *f*–*I* curves generated from distal current inputs at the same time as the somatically induced *f*–*I* traces (see also Clopath et al., 2007). One could potentially maintain the closed-form tractability of the model in this scenario by first fitting *f*–*I* curves from different dendritic injection sites separately, and then combining them into the same model by optimizing parameters of the transfer functions. One other obvious limitation already pointed out is that the model is not able to capture phenomena resulting from the progressive inactivation of Na^+^ channels at higher potentials like the depolarization block. Neither the approximation to the AdEx nor the AdEx itself are able to capture the sudden decrease or non-monotonicity in firing rates as the driving current becomes very high, since they lack a bifurcation from the stable limit cycle associated with periodic spiking to a stable fixed point (in contrast to other simple neuron models, e.g., Izhikevich, 2007; Durstewitz, 2009). The phenomenon of depolarization block itself may simply be captured by introducing a current threshold beyond which spiking is shut off, but of course this would not capture more subtle changes in the neuron’s behavior resulting from progressive inactivation of inward currents. However, as already discussed above, extensions of the model along this direction may be less relevant if the main goal is to capture network-dynamical regimes that are presumably characteristic of awake *in vivo* scenarios (balanced state).

To conclude, we derived an approximation to the AdEx model which allows for fast fitting to physiological data while retaining most of the dynamical features of the original model. Our model fitting approach relies entirely on *f*–*I* and *I*–*V* curves as obtained routinely in standard electrophysiological protocols, yet the resulting models predict reasonably well spike times and behavior obtained with *in vivo*-like fluctuating-current inputs not used for model fitting. Thus this approach may allow to efficiently and automatically translate, in a kind of “high-throughput” fashion, larger data bases of single-cell recordings into a computational framework. It can be used to construct networks of in this sense physiologically validated model cells which are still computationally efficient and analytically tractable.

## Conflict of Interest Statement

The authors declare that the research was conducted in the absence of any commercial or financial relationships that could be construed as a potential conflict of interest.
